# Radiation Shielding Tests of Crosslinked Polystyrene-b-Polyethyleneglycol Block Copolymers Blended with Nanostructured Selenium Dioxide and Boron Nitride Particles

**DOI:** 10.3390/nano12030297

**Published:** 2022-01-18

**Authors:** Zehra Merve Cinan, Burcu Erol, Taylan Baskan, Saliha Mutlu, Bülend Ortaç, Sevil Savaskan Yilmaz, Ahmet Hakan Yilmaz

**Affiliations:** 1 Faculty of Sciences, Department of Physics, Karadeniz Technical University, Trabzon 61080, Turkey; m_cinan@ktu.edu.tr (Z.M.C.); taylanbaskan@ktu.edu.tr (T.B.); 2 Faculty of Arts and Sciences, Department of Physics, Recep Tayyip Erdoğan University, Rize 53100, Turkey; burcu.karayunus@erdogan.edu.tr; 3 Faculty of Sciences, Department of Chemistry, Karadeniz Technical University, Trabzon 61080, Turkey; salihamutlu@ktu.edu.tr (S.M.); sevily@ktu.edu.tr (S.S.Y.); 4 National Nanotechnology Research Center and Institute of Materials Science and Nanotechnology, Bilkent University, Ankara 06800, Turkey; ortac@unam.bilkent.edu.tr

**Keywords:** attenuation characteristics, boron nitride, gamma shield, nanocomposite, polystyrene-b-polyethyleneglycol, selenium dioxide, protection material

## Abstract

In this work, gamma-ray shielding features of crosslinked polystyrene-b-polyethyleneglycol block copolymers (PS-b-PEG) blended with nanostructured selenium dioxide (SeO_2_) and boron nitride (BN) particles were studied. This research details several radiation shielding factors i.e., mass attenuation coefficient ($\mu_{m}$), linear attenuation coefficient ($\mu_{L}$), radiation protection efficiency (RPE), half-value layer (HVL), tenth-value layer (TVL), and mean free path (MFP). The irradiation properties of our nanocomposites were investigated with rays from the ^152^Eu source (in the energy intervals from 121.780 keV to 1408.010 keV) in a high-purity germanium (HPGe) detector system, and analyzed with GammaVision software. Moreover, all radiation shielding factors were determined by theoretical calculus and compared with the experimental results. In addition, the morphological and thermal characterization of all nanocomposites was surveyed with various techniques i.e., nuclear magnetic resonance (NMR), Fourier-transform infrared spectroscopy (FTIR), thermogravimetric analysis (TGA), scanning electron microscopy (SEM), energy-dispersive X-ray spectroscopy (EDX), and transmission electron microscopy (TEM). Acceptable compatibility was revealed and observed in all nanocomposites between the experimental and theoretical results. The PS-b-PEG copolymer and nanostructured SeO_2_ and BN particles exerted a significant effect in enhancing the resistance of the nanocomposites, and the samples with high additive rates exhibited better resistance than the other nanocomposites. From the achieved outcomes, it can be deduced that our polymer-based nanocomposites can be utilized as a good choice in the gamma-irradiation-shielding discipline.

## 1. Introduction

In many areas of life, we are exposed to radiation—both naturally, and as a result of the facilities provided by technological innovations. Human bodies and systems can be influenced by radiation in ways that we are not aware of. While these influences produce observable results in some cases, radiation occasionally penetrates our bodies without our awareness. Radiation is an increasingly common phenomenon on Earth. Radio waves that enable radio and television communication, X-rays used in medicine and industry, cosmic rays, etc., are the types of radiation we are accustomed to in daily life. With the advancement of technology and industrialization bringing the utilization of radioactive sources in various fields of study, the harmful effects of radiation have gradually increased. Nuclear technology has applications in fields such as medicine, scientific research, agriculture, industry, and archaeometry—and especially in the energy sector. Nuclear energy has significant advantages compared to other types of energy; it can be said that the most important of these advantages is the ability to obtain high energy production per unit mass. Despite its advantages, waste control, initial investment cost, and radiation safety are among its most prominent disadvantages.

Radiation comes first among the tools used in the examination and development of the characteristics of substances. Examining and enhancing the characteristics of materials are among the main objectives of science and industry. In this context, examining the interaction between radiation and matter is of great significance in terms of science and technology. With the development of nanotechnology, the transition from microstructure to nanostructure has manifested itself, and the nanostructural properties of materials have begun to be determined [1,2,3,4,5]. Radiation sources used to obtain information on the nanostructure of materials include X-rays, gamma rays, neutrons, electrons, protons, and high-energy ions. In this context, it is important to provide information about radiation types and structures in order to reveal the interaction of radiation with the material.

Revealing the nanostructural properties of materials leads to the expansion of the use of materials in industry. In addition, it is possible to develop material properties in line with the determined purposes, and to provide different usage conditions in various industries. Thus, many outputs—such as ease of energy production, high efficiency, and high security—can be obtained. The fields in which the acquired knowledge is put into practice include the aerospace industry, the energy sector, nuclear technology, electronic/computer applications, construction, mining, and medicine.

With the advances in technology, the properties of materials alone have become insufficient in their use. Different materials can be combined, enabling their use for different purposes. When these materials—called composite materials—are brought together, they have more advanced properties than the materials of which they are composed. In nuclear technology, as in other industries, composite materials are widely used [6,7,8,9,10]. Composite materials are also used for many other purposes—especially radiation safety. Among the main objectives of radiation security is to protect humans, tissues, and the perimeter from the unfavorable impacts of radiation. Protection from the effects of radiation includes two conditions: protection under normal and abnormal conditions. Under normal conditions, the impermeability of radioactive and radiation-containing materials is the main objective, while under abnormal conditions, the flexibility, durability, high corrosion resistance, and thermal properties of the materials are taken into account, in addition to the former [11,12].

Many studies investigating the structure of radiation shielding materials have been and continue to be conducted [13,14,15,16,17,18]. Polymeric substances have been an important point of research-based concern in various multidisciplinary and commercial works, because of their valuable characteristics (lightness, flexibility, high strength, thermal stability, resistance to corrosion, durability, etc.) and capabilities. For this reason, researchers have remarkably focused on polymer-based irradiation (gamma, electromagnetic, etc.) shielding substances [11,12,19,20,21,22]. To develop an effective neutron shielding material, Zhang et al. worked on alternating multilayered composites (high-density polyethylene/high-density polyethylene/boron nitride (HDPE/HDPE/BN) and HDPE/BN/HDPE/barium sulfate (BaSO_4_) systems), and found that neutron permeability noticeably diminished with multilayered composites, and that compared to classic polymer-based substances, these composites illustrated great shielding performance [19]. Mahmoud et al. studied a recycled high-density polyethylene (R-HDPE) with phosphotungstic acid (PTA) and copper oxide nanoparticles (CuO NPs) to construct shielding against gamma irradiation, and found that their nanocomposite materials were extremely preventive against γ-rays because of the existence of high-density substances, such as PTA and CuO nanoparticles [20]. Al-Burahi et al. surveyed the radiation shielding characteristics of several mercantile polymers—such as poly(N-isopropyl acrylamide), polyethylene terephthalate, polystyrene, and polycarbonate—and suggested a prospective use of the investigated mercantile polymers as up-and-coming materials for radiation protection implementations [21]. Mortazavi et al. aimed to develop a radiation shield for application against both neutron and gamma radiation; for this purpose, they developed borated polyethylene nanocomposite materials, and found that the best attenuation was obtained from a borated polyethylene nanocomposite material that was 5 wt% boron-doped [22]. Olukotun et al.’s research focused on a self-maintaining hydrogenous clay/polyethylene-based composite for use as a shielding material [23]; their results showed that this material has enriched shielding properties. Alabsy et al. investigated frequently used polymers in clinical implementations, novel polymers, and their interaction with radiation via simulation [24]; their research deduced that these polymer structures can be utilized with some high-Z fillers as radiation shields. More et al.’s review research focused on the synthesis and efficacy of polymeric-based substances blended with nano-additives for radiation safeguarding [25]; furthermore, they introduced the recycling of polymer structures. Almurayshid et al. suggested that polymer-based shielding materials (e.g., ethylene vinyl acetate (EVA) polymers, Si, SiC, B_4_C) offer a mild, low-cost, nontoxic means of annihilating the harmful effects of generated neutrons, and the conclusions of their research illustrated that the blended materials (i.e., Si, SiC, B_4_C) in the polymer enhanced the attenuation ability in low-energy regions [26]. Muthamma et al. aimed to investigate the gamma shielding properties of diglycidyl ether of bisphenol A (DGEBA) epoxy-resin-based micro- and nano-Bi_2_O_3_ composites, and they found that nanocomposites have preferable gamma attenuation at all energy regions (from 0.356 to 1.332 MeV), compared to microcomposites with identical doping; furthermore, this work elicited the importance of nanostructural additives to improve the routine activities of the composites [27]. Malinowski et al. worked on the composition and mechanical characteristics of hemp-fiber-reinforced poly(ε-caprolactone) samples reformed via electron beam irradiation [28], and they observed that these materials can be used for the manufacture of materials, depending on the amount of radiation and the fiber contribution. Güven’s research focused on radiation-supported production of polymeric nanomaterials, suggesting that these developed materials with high added value will not be limited to existing applications, but will be enriched with different usage properties [29]. Acevedo-Del-Castillo et al. investigated the use of polymer nanocomposites to attenuate the implementation of high-energy irradiation, and found that the increase in the attenuating characteristics of slight, resilient, and well-rounded substances (such as polymeric materials) can be a contribution of upcoming technologies to the acquisition of more influential substances for diminishing the detriment caused to the high-energy electromagnetic radiation (HE-EMR) in several areas where it is found [30]. Doyan et al. worked on the polymeric film materials of polyvinyl alcohol (PVA), trichloroethylene (TCE), and cresol red (CR) dye to expose the gamma beams for eventual implementation in radiation dosimetry; their results indicate that enhancement of the radiation rate altered the hue of the polymeric film, from purple (pH > 8.8) with no radiation (0 kGy) to yellow (nearly pellucid) (2.8 < pH < 7.2) at the uppermost rate (12 kGy); in other words, these results demonstrated a useful dose range of 0–12 kGy for the investigated polymeric film samples [31]. Smolyanskii et al. worked on the construction of polytetrafluoroethylene developed via the combination of gamma irradiation and high temperatures, and concluded that these conditions (327–350 °C) lead to the evanescence of the poriferous texture and the creation of a few large pores [32]; furthermore, they indicated that it is important to carry out new research on the continuity of changes in the structure and irradiation–chemical operations, depending on the irradiation temperature. A. Abu Saleem et al. focused on the shielding properties of epoxy + carbon- and glass-fiber composites doped with lead nanoparticles [33]; their conclusions indicate that the $\mu_{m}$ values rise as lead nanoparticle rates increase up to a specific proportion (~15 wt%), after which the enhancement in the $\mu_{m}$ values becomes negligible.

Selenium (Se) is a non-metallic element—an insider of group XVI of the periodic table—and is generally used to cultivate corrosion durability. Furthermore, selenium is a common volatile element in coal and minerals [34,35], and has been classified as one of the most volatile trace elements (TEs) by Clarke and Sloss [36]. Selenium nanoparticles (Se NPs), with lower toxicity and better attenuation capacity than inorganic and organic modes, have a comprehensive range of implementations in medicinal diagnostics and nanobiotechnology [37]. Synthesized Se NPs are receiving increasing attention because of their special physical, chemical, and biological properties [38].

Elshami et al. worked on the radiation shielding qualification of neo-improved PbO-B_2_O_3_-SeO_2_-Er_2_O_3_:Au_2_O_3_ glass–ceramic [(40)PbO-(10)B_2_O_3_-(49.50; 49.25; 49.00; 48.75; 48.50)SeO_2_-(0.5)Er_2_O_3_: (0; 0.025; 0.050; 0.075; 0.1)Au_2_O_3_] materials, and found that the attenuation coefficients had 0.233 cm^2^/g distinctions among the various Au_2_O_3_-doped substances; they deduced that the researched ceramics had good shielding qualifications to utilize for several implementations of gamma irradiation [39]. Kebaili et al. worked to investigate the gamma and beta radiation shielding properties of neo-improved 60Se-20Ge(20−x)-Sb-xAg (x values ranging from 0 to 20 mol%) chalcogenide glass structures; when they compared the improved glass structures with mercantile glass structures and conventional concrete, they concluded that the results show a good possibility of using the investigated substances in radiation shielding implementations [40]. El-Qahtani et al. worked on the use of chalcogenide glass materials as shielding materials for extremely potent forms of radiation (i.e., X-rays, gamma rays, and neutrons), and investigated As_40_-Se_60−x_-Sn_x_ (x changes from 0% to 20% with 5% steps) chalcogenide glass structures; they concluded that their explored system’s results illustrate better prevention of neutron and gamma irradiation than some standard and mercantile glass structures [41]. Kebaili et al. suggested that the investigated glass structures (Ge_20_Se_80−x_Bi_x_ (x ≤ 12)) have potential as practical and effective shielding materials [42].

Materials designed using different components to absorb radiation have been examined in detail in different energy spectra, and revealing the characteristics at all energies and wavelengths has been the main focus of many researchers [43,44,45,46,47,48,49].

In this study, we aimed to handle the gamma-ray shielding features of PS-b-PEG blended with nanostructured selenium dioxide (SeO_2_) and boron nitride (BN) particles and to reduce the harmful effects of radiation. For this purpose, our work delineates several radiation shielding factors (i.e., $\mu_{m}$, $\mu_{L}$, RPE, HVL, TVL, and MFP, etc.). The irradiation properties of our nanocomposites were investigated with rays from the ^152^Eu source (in the gamma-ray intervals from 121.780 keV to 1408.010 keV) in a high-purity germanium (HPGe) detector system (ORTEC-AMETEK, Oak Ridge, TN, USA) and analyzed with GammaVision software (ORTEC-AMETEK, Oak Ridge, TN, USA). Moreover, all radiation shielding factors were determined by theoretical calculus and compared with the experimental results [50,51,52,53]. Since the interactions of rays of different energies and wavelengths with atoms in the matter differ, the ^152^Eu radioactive source was used to present the most comprehensive results. The ^152^Eu, which was the radioactive source used in the examination, provides the opportunity to examine samples in the widest variety of energy ranges.

Acceptable compatibility was observed in all nanocomposites between the experimental and theoretical results. The PS-b-PEG copolymer and nanostructured SeO_2_ and BN particles played a significant role in enhancing the resistance of the nanocomposites, and the samples with high additive rates exhibited better resistance than the other nanocomposites. From the achieved outcomes, it can be deduced that our polymer-based nanocomposites can be utilized as a good choice for use in the gamma-irradiation-shielding discipline in many areas (such as flexible and durable gamma-radiation-protective systems for the transportation of radioactive materials, isolation for radioactive waste operations, and radiation services in hospitals, nuclear power plants (NPPs), the defense industry, the building industry, and many others).

## 2. Material and Methods

### 2.1. Synthesis and Characterization

**Scheme 1:**
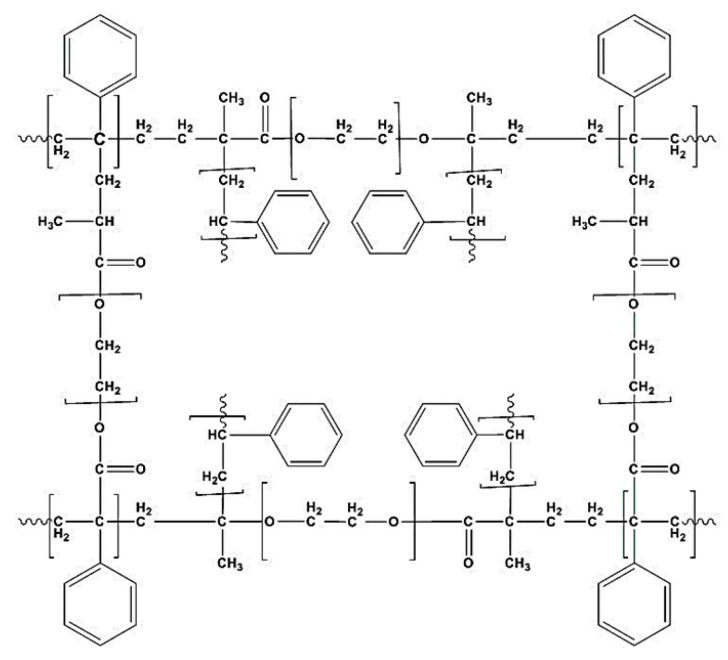
The reaction pathways of the crosslinked polystyrene-b-polyethyleneglycol block copolymers (PS-b-PEG).

The synthesis, characterization, and detailed analysis of the PS-b-PEG in all nanocomposites used in this investigation were carried out at our research group’s laboratory [12,54,55,56,57]. All PS-b-PEG copolymers (1000, 1500, and 10,000) included in this study’s nanocomposites were crosslinked. The FTIR and NMR attributes obtained for the characterization of the PEG (1000-1500-10,000) crosslinkers, the PS-b-PEG copolymers, and the characteristic peaks of the polymers are detailed in [12,54,55,56,57]. The reaction pathways of this copolymer structure can be viewed in Scheme 1.

### 2.2. Characterization of Polymer–Nanostructured-Particle-Based Nanocomposites

#### 2.2.1. Thermogravimetric Analysis (TGA)

The TGA outcomes of the PS-b-PEG copolymers blended with the nanostructured SeO_2_ and BN particles were measured with the Seiko II Exstar 6000 (Seiko Instruments Inc., Chiba-shi-Chiba, Japan) TGA/DTA analyzer system.

#### 2.2.2. Scanning Electron Microscopy (SEM) and Energy-Dispersive X-ray Spectroscopy (EDX) Examinations

Scanning electron microscopy (SEM) analyses were carried out using a Quanta 200 FEG-SEM (field-emission gun (FEG)-scanning electron microscope (SEM)) (FEI Company, Hillsboro, OR, USA). Before SEM analyses, nanocomposites were fixed on a carbon tape and coated with ~10 nm Au/Pd alloy (with PECS-682) in order to prevent the electron-charging effect. The energy-dispersive X-ray (EDX) analysis was also conducted for the constitutional explanation of the nanocomposites.

#### 2.2.3. Transmission Electron Microscopy (TEM) and EDX Examinations

The structural analyses of polymer–nanostructured-particle-based nanocomposites were carried out using a transmission electron microscope (TEM) (FEI-Tecnai G2F30, Hillsboro, OR, USA) system equipped with an EDX. A scanning transmission electron microscope (STEM)-EDX was used to examine SeO_2_ nanoparticles on polymer nanostructures. TEM samples were also prepared by fixing nanomaterials onto carbon-coated TEM grids.

### 2.3. Preparation of Polymer–Nanostructured-Particle-Based Nanocomposites

Table 1 exemplifies the formation of each of the developed nanocomposites. The templates of these nanocomposites were constructed at a temperature range of 20–22 °C, via pressing under 10 MPa stress for 15–25 min. The templates had a caliber of 12–13 mm, and their diameters were gauged using a BTS 12051 external micrometer (with a reading sensitivity from 0.01 mm to 25 mm).

### 2.4. Experimental Framework for Gamma Irradiation of Polymer–Nanostructured-Particle-Based Nanocomposites

**Scheme 2:**
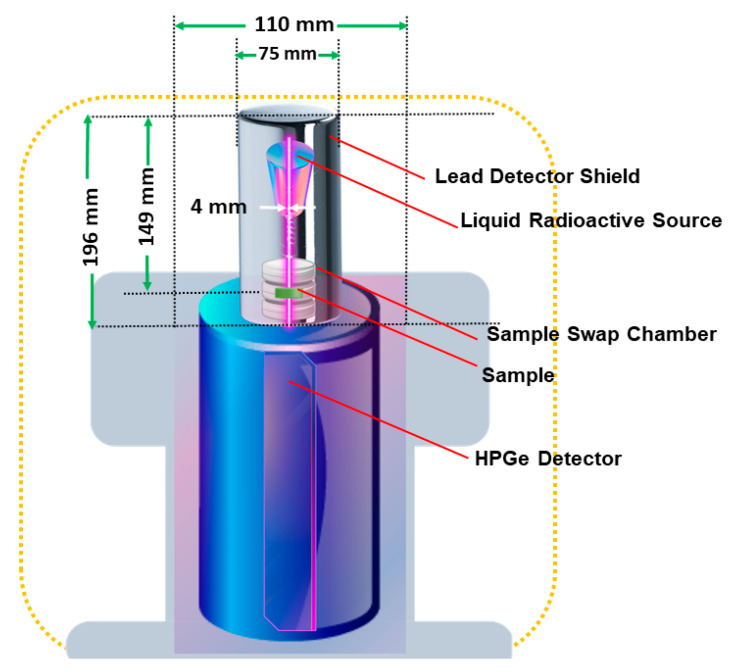
Diagram of the HPGe detector for gamma irradiation attenuation experiments.

The irradiation properties of the nanocomposites were explored via a spacious-interval gamma-ray spectrum with the ^152^Eu source (in the gamma-ray intervals from 121.780 keV to 1408.010 keV) in an HPGe detector system (ORTEC-AMETEK, Oak Ridge, TN, USA), and analyzed with GammaVision software (ORTEC-AMETEK, Oak Ridge, TN, USA); the experimental setup is portrayed in Scheme 2. Moreover, all radiation shielding factors (Table 2) were determined by theoretical calculus and compared with the experimental results.

The $I_{0}$, I, x, $w_{i}$, and ρ symbols in Table 2 refer to the non-attenuated photon densities, attenuated photon density, nanocomposite material thickness, weight portion, and the density of the nanocomposites, respectively.

## 3. Results and Discussions

### 3.1. Characterization of Polymer–Nanostructured-Particle-Based Nanocomposites via SEM, TEM, and EDX Systems

#### 3.1.1. Characterization of Polymer–Nanostructured-Particle-Based Nanocomposites via SEM

The morphological characterization of all nanocomposites was surveyed using the scanning electron microscopy (SEM) and energy-dispersive X-ray spectroscopy (EDX) techniques; the results are summarized in this section.

**Figure 1:**
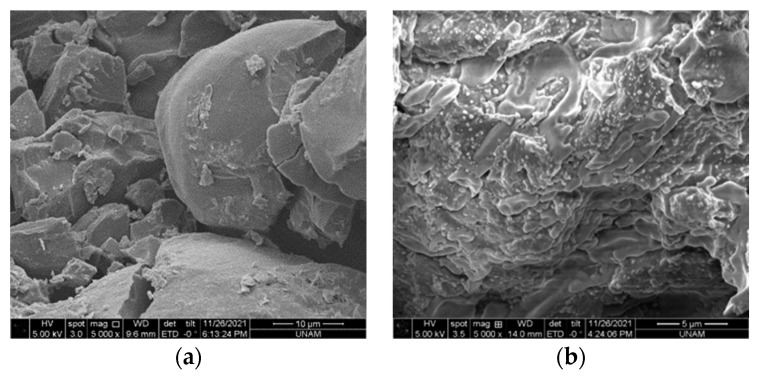
SEM images of PSNC1 (**a**) and PSNC4 (**b**) nanocomposites.

**Figure 2:**
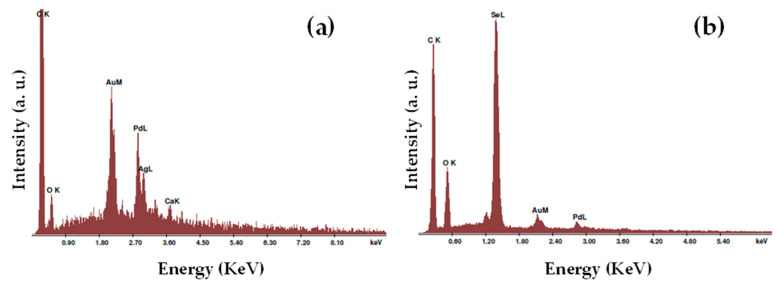
EDX spectra of PSNC1 (**a**) and PSNC4 (**b**) nanocomposites.

From the SEM photographs of the PSNC1 nanocomposite (Figure 1a and Figure S1a,b), it was found that the polymer surface morphology was grainy and rough, and there were accumulations on the surface. When SeO_2_ nanoparticles were added to PSNC1, it was observed that the PSNC4 nanocomposite had a granular structure similar to the morphology of the PSNC1 polymer, and the SeO_2_ nanoparticles were dispersed as small particles on the surface (Figure 1b and Figure S1c,d). The EDX graphics in Figure 2b show the binding energy of the SeO_2_ nanoparticles to be 1.45 keV, supporting the existence of the SeO_2_ nanoparticles in the composite. In the EDX graph in Figure 2a, no SeO_2_ nanoparticles were observed inside the PSNC1 polymer.

**Figure 3:**
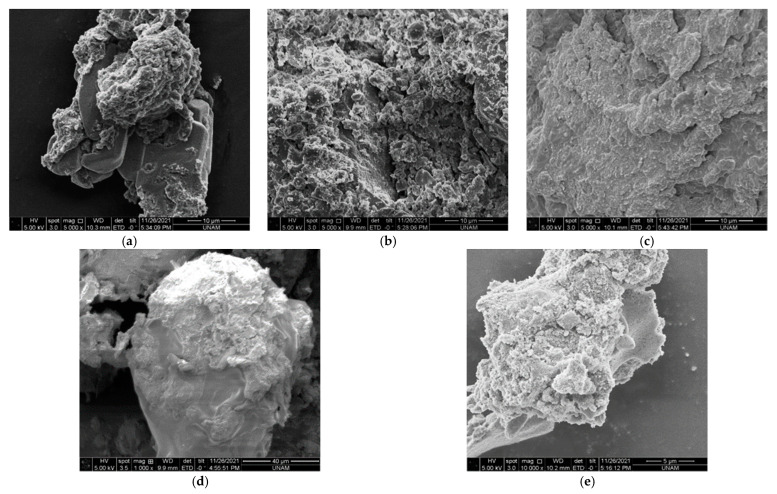
SEM images of PSNC9 (**a**), PSNC14 (**b**), PBSNC2 (**c**), PBSNC6 (**d**), and PBSNC10 (**e**) nanocomposites.

When SEM images of the PSNC9 nanocomposite are examined, it can be seen that the surface morphology is rough and granular, and very small SeO_2_ nanoparticles are located on the surface (Figure 3a and Figure S2a,b). SEM photographs of the PSNC14 nanocomposite containing SeO_2_ also show a morphology similar to that of PSNC9 (Figure 3b and Figure S2c,d).

When BN nanoparticles were added to the polymer/SeO_2_ nanocomposite (for PBSNC2, PBSNC6, and PBSNC10 nanocomposites), it was observed that the BN nanoparticles adhered to the surface very well, along with the SeO_2_ nanoparticles, and their distribution was homogeneous (Figure 3c–e and Figure S2e–j). As a result, it can be seen that as the molecular weight of the macro-crosslinker increases, the polymer particles become more pronounced, and the nanoparticles are dispersed on the surface.

#### 3.1.2. Characterization of Polymer–Nanostructured-Particle-Based Nanocomposites via TEM

The morphological characterization of all nanocomposites was surveyed using the transmission electron microscopy (TEM) and energy-dispersive X-ray spectroscopy (EDX) techniques.

**Figure 4:**
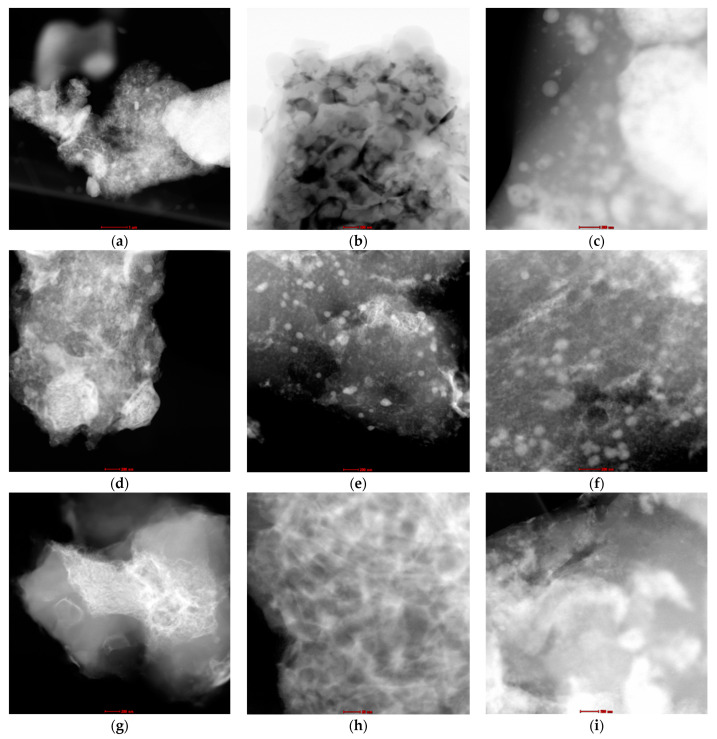
TEM images of PSNC14 (**a**–**c**), PSNC4 (**d**–**f**), and PBSNC6 (**g**–**i**) nanocomposites.

When the TEM images of the PSNC4 nanocomposite are compared with the SEM images (Figure 1a,b and Figure S1a–d), it can be seen that the SeO_2_ nanoparticles added to the PSNC4 polymer are more prominently dispersed in the interior of the polymer (Figure 4a–c).

**Figure 5:**
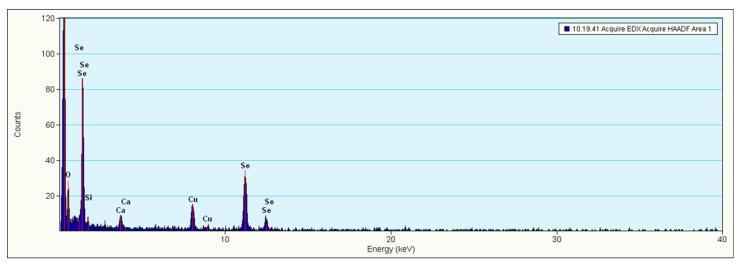
EDX spectrum of the PSNC14 nanocomposite.

When the TEM images of the PSNC14 nanocomposite are examined, it can be seen that as the molecular weight of the macro-crosslinker in the PSNC14 composite increases, unlike the PSNC4 nanocomposite, the particle density of the SeO_2_ nanoparticles added to the surface decreases, and the nanoparticles enter the pores (Figure 4d–f). SeO_2_ peaks appearing at 2 keV and 10–12.5 keV binding energies in the EDX graph of the PSNC14 nanocomposite (Figure 5) show that there is SeO_2_ distribution in different layers in the polymer.

When BN nanoparticles were added to the polymer/SeO_2_ nanocomposite (for the PBSNC6 nanocomposite), the BN nanoparticles visibly changed the distribution and particle structure of the SeO_2_ nanoparticles in the composite (Figure 4g–i).

### 3.2. Thermogravimetric Analysis (TGA) Outcomes of the PS-b-PEG Copolymers Blended with the Nanostructured SeO_2_ and BN Particles

**Figure 6:**
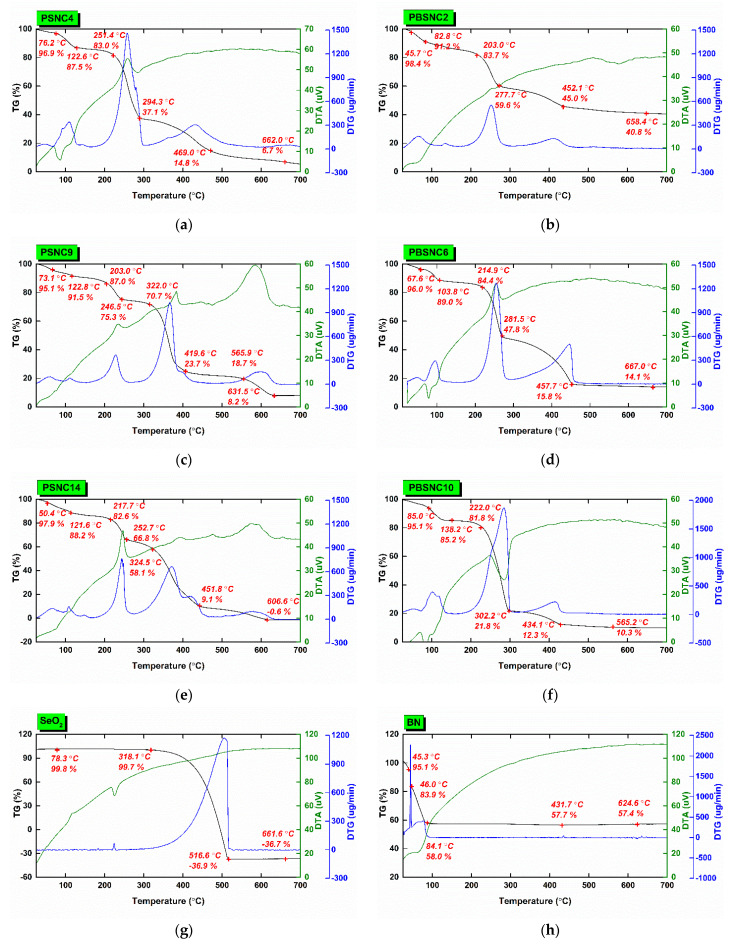
TGA outputs of the PS-b-PEG copolymers blended with the nanostructured SeO_2_ and BN particles—PSNC4 (**a**); PBSNC2 (**b**); PSNC9 (**c**); PBSNC6 (**d**); PSNC14 (**e**); PBSNC10 (**f**)—and TGA outputs of the pure nanostructured SeO_2_ and BN particles: SeO_2_ (**g**); BN (**h**).

In Figure 6a–f, the TGA curves of the PS-b-PEG copolymers blended with the nanostructured SeO_2_ and BN particles are exhibited in detail, and wt% loss rates in some of our nanocomposites at miscellaneous temperatures are presented in Table S1 (available in the Supplementary Materials). It can be deduced that these TGA curves represent thermogravimetric impairments of the SeO_2_- and BN-filled PS-b-PEG-based nanocomposites that were analyzed in the temperature range of 45.7–667.0 °C.

In Figure 6a and Table S1 (available in the Supplementary Materials), the temperature at which the PSNC4 nanocomposite including 10% PS-b-PEG (1000) and 90% nanostructured SeO_2_ particles starts to deteriorate is 76.2 °C; 3.1% decrement was experienced up to this temperature value. In addition, a decrease of 4.5% was observed between 122.6 °C and 251.4 °C. The next degradation began at 251.4 °C, and proceeded to 294.3 °C; in other words, a 45.9% decrease was experienced up to this point. This change continued until 662.0 °C. There was a decrease of 30.4% from 294.3 °C until this temperature. When we changed the PS-b-PEG (1000) ratio to 15% and the ratio of the SeO_2_ particles to 70%, and added 15% nanostructured BN particles, the temperature at which the PBSNC2 nanocomposite first started to decompose was 45.7 °C, and there was a 7.2% loss between 45.7–82.8 °C. The degradation lasted until 203.0 °C, and there was a 7.5% loss in this range, as shown in Figure 6b. The next degradation commenced at 203.0 °C and continued until 277.7 °C, decreasing by 24.1% in this range. The final decomposition started at 277.7 °C, and lasted from 452.1 °C to 658.4 °C, decreasing by 18.8% in this interval. The decomposition percentage was similar to that viewed from the thermogram between 452.1 and 658.4 °C.

The temperature at which the PSNC9 nanocomposite including 10% PS-b-PEG (1500) and 90% nanostructured SeO_2_ particles started to degrade was 73.1 °C, and there was a 4.9% loss up to this temperature. The percentage rates close to one another from 122.8 °C to 203.0 °C were observed from the thermogram as 91.2–83.7%. The second impairment commenced at 203.0 °C and continued from 246.5 °C to 322.0 °C, decreasing by 16.3% at this interval, as shown in Figure 6c. The most apparent impairment in this TGA can be observed between 322.0 and 419.6 °C—there was a 47.0% loss in this interval. From 419.6 °C, the final degradation lasted until 631.5 °C, decreasing by 15.5% in this range. When we increased the PS-b-PEG (1500) ratio to 15%, reduced the ratio of the SeO_2_ particles to 70%, and added 15% nanostructured BN particles, the temperature at which the PBSNC6 nanocomposite first started to decompose was 67.6 °C, and there was a 4.0% loss at this temperature. As can be seen in Figure 6d, the percentage rates close to one another from 103.8 °C to 214.9 °C were observed from the thermogram as 89.0–84.4%. The most apparent impairment in this TGA curve can be observed between 214.9 and 281.5 °C—there was a 36.6% loss in this interval. The next degradation commenced at 281.5 °C and continued to 457.7 °C, decreasing by 32.0% in this range. The final degradation lasted from 457.7 °C to 667.0 °C; the near-constant rate can be read from the thermogram as 15.8–14.1% in Table S1 (available in the Supplementary Materials).

The temperature at which the PSNC14 nanocomposite including 10% PS-b-PEG (10,000) and 90% nanostructured SeO_2_ particles first started to decompose was 50.4 °C, lasting from 121.6 °C to 217.7 °C, decreasing by 17.4% in this interval. The following deterioration began at 217.7 °C, and lasted from 252.7 °C to 324.5 °C, decreasing by 24.5% in this interval. In Figure 6e and Table S1, it can be seen that the final degradation lasted from 324.5 °C to 606.6 °C; the loss percentage can be read from the thermogram as 58.7%. As can be seen in Figure 6f and Table S1, when we altered the PS-b-PEG (10,000) ratio to 15% and the ratio of the SeO_2_ particles to 70%, and added 15% nanostructured BN particles, the temperature at which the PBSNC10 nanocomposite’s first decomposition began was 85.0 °C, and 4.9% decline was experienced up to this value. There was a 13.3% loss between 85.0–222.0 °C. The next and most clear degradation in this TGA curve can be read between 222.0–302.2 °C—there was a 60.0% loss in this region. The final degradation lasted from 302.2 °C to 565.2 °C; an 11.5% decrement was experienced up to this temperature value, and the near-constant rate can be read from the thermogram as 12.3–10.3% between 434.1 and 565.2 °C in Table S1.

Figure 6g,h represent the TGA outputs of the pure nanostructured SeO_2_ and BN particles under heat treatment. In Figure 6g, for the pure nanostructured SeO_2_ particles, the weight seems to indistinctly reduce between 78.3 and 318.1 °C in Table S1. The next and most clear degradation in this TGA can be observed between 318.1 and 516.6 °C; the degradation is nearly the same between 516.6 and 661.6 °C. In Figure 6h, for the pure nanostructured BN particles, the first decomposition starts at 45.3 °C, and lasts from 46.0 °C to 84.1 °C, decreasing by 37.1% in this interval; the degradation is nearly the same between 431.7 and 624.6 °C.

Cinan et al. observed that for pure PS-PEG (1000), initial sample degradation began at 46.7 °C, and there was an 11.380% reduction between 46.7 and 91.0 °C. The real degradation began at 254.2 °C, with 36.380% diminution by 381.2 °C. The pure PS-PEG (1500) sample began to deteriorate at 53.4 °C. The rates between 53.4 and 322.4 °C were observed as 93.500–82.500%, then again started to change, from 82.500% at 322.4 °C to 0.500% at 417.9 °C. The pure PS-PEG (10,000) sample began to deteriorate at 31.2 °C, with 20.040% reduction observed around 31.2–66.5 °C. The temperatures between 66.5 and 293.8 °C were observed as 76.020–69.250%. This sample again started to vary, from 69.250% at 293.8 °C to 3.000% at 401.6 °C, with a deterioration rate of 66.250% between 293.8 and 401.6 °C [12].

The TGA curves of our nanocomposites were exhaustively analyzed to characterize the decomposition of the PS-b-PEG copolymers blended with the nanostructured SeO_2_ and BN particles. When we compare the thermograms in detail, the peaks at 250–400 °C, 400–550 °C, and 0–150 °C can be ascribed to the PS-PEG copolymers, nanostructured SeO_2_, and BN particles, respectively. In all thermograms, there were few differences in the nanocomposites when heated to ~700.0 °C, demonstrating that our PS-b-PEG-based nanocomposites were thermally balanced. The TGA curves of our PS-b-PEG nanocomposites illustrate the decomposition of the PS-b-PEG copolymers blended with the nanostructured SeO_2_ and BN particles. As can be seen from all TGA curves, the applied gamma radiation does not influence the construction of the developed PS-b-PEG-based nanocomposites. To summarize all of the results, four thermal disintegration regions were monitored around 70 °C, 200 °C, 450 °C, and 650 °C. The first changes started at around 70.0–80.0 °C, and the last degradation around 660.0 °C led to the thermal disintegration of the PS-b-PEG (1000) nanocomposites. The other TGA curves of our nanocomposites (for the PS-b-PEG (1500) and the PS-b-PEG (10,000)) illustrated thermal decomposition characteristics parallel to those of the PS-b-PEG (1000) nanocomposites. In the six TGA thermograms, it can be observed that as the temperature increases there is a decrease in weight %, thus showing that mass is altering slightly as a result of thermal behavior. In the thermograms, the first degradation begins at a nominal temperature, and proceeds up to a higher temperature with changes in weight rates. Consequently, the nanocomposites with higher percentages of nanostructured SeO_2_ and BN particles represented preferable thermal uniformity, because the greater percentage of nanostructured particles acted like walls, protecting the chains formed on the surface from decomposition.

### 3.3. Gamma Irradiation Results

#### 3.3.1. Linear ($\mu_{L}$) and Mass Attenuation ($\mu_{m}$) Coefficients

**Figure 7:**
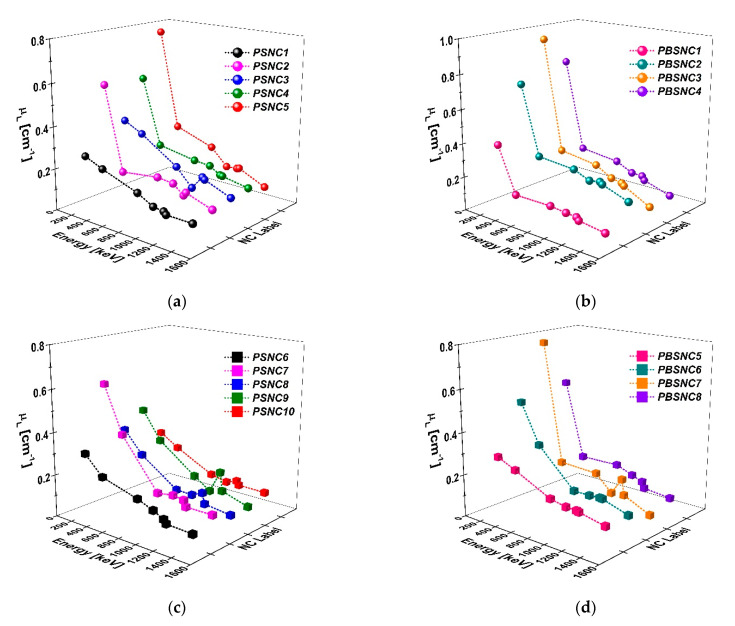

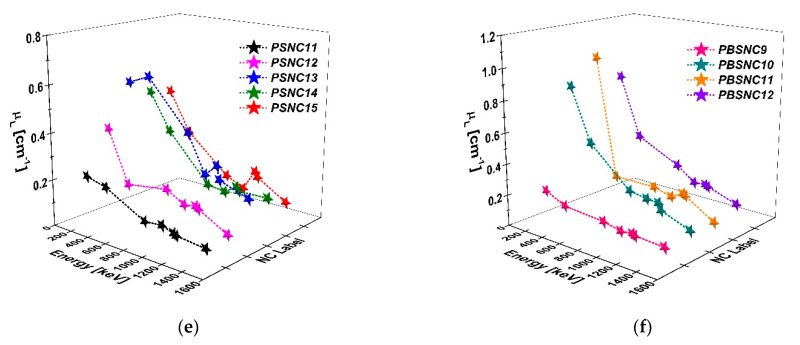
The $\mu_{L}$ rates of the PS-b-PEG copolymers blended with the nanostructured SeO_2_ and BN particles under a wide range of gamma irradiation energies: PSNC1, PSNC2, PSNC3, PSNC4, and PSNC5 (**a**); PBSNC1, PBSNC2, PBSNC3, and PBSNC4 (**b**); PSNC6, PSNC7, PSNC8, PSNC9, and PSNC10 (**c**); PBSNC5, PBSNC6, PBSNC7, and PBSNC8 (**d**); PSNC11, PSNC12, PSNC13, PSNC14, and PSNC15 (**e**); PBSNC9, PBSNC10, PBSNC11, and PBSNC12 (**f**).

**Figure 8:**
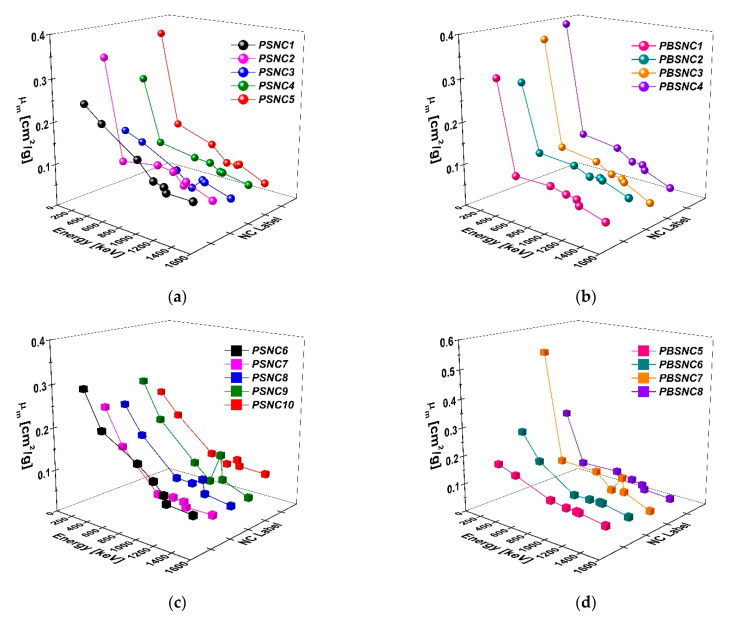

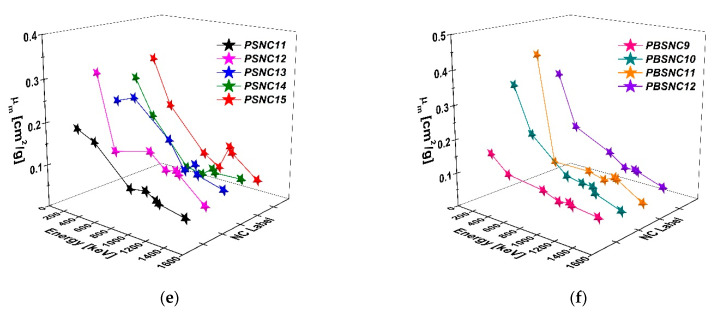
The $\mu_{m}$ rates of the PS-b-PEG copolymers blended with the nanostructured SeO_2_ and BN particles under a wide range of gamma irradiation energies: PSNC1, PSNC2, PSNC3, PSNC4, and PSNC5 (**a**); PBSNC1, PBSNC2, PBSNC3, and PBSNC4 (**b**); PSNC6, PSNC7, PSNC8, PSNC9, and PSNC10 (**c**); PBSNC5, PBSNC6, PBSNC7, and PBSNC8 (**d**); PSNC11, PSNC12, PSNC13, PSNC14, and PSNC15 (**e**); PBSNC9, PBSNC10, PBSNC11, and PBSNC12 (**f**).

The $\mu_{L}$ and $\mu_{m}$ values from the 121.782 keV to 1408.006 keV radiation energy regions are demonstrated in Figure 7 and Figure 8 and Tables S2–S5) (the tables are present in the Supplementary Materials). It can be observed that the gamma attenuation characteristics give good outcomes for all nanocomposites in ranges within the chosen gamma irradiation energy interval. Furthermore, it can also be seen in all graphs that it is somewhat more challenging to attenuate gamma rays in the high-energy regions than in the low-energy regions.

There was satisfactory accordance determined between the theoretical and experimental $\mu_{L}$ values of the researched nanocomposites. When we examine the linear attenuation behaviors, the success in shielding against gamma rays is obvious for both of the PS-b-PEG copolymers blended with the nanostructured SeO_2_ and BN nanocomposites, as shown in Figure 7 and Tables S2 and S3. Specifically, the PS-b-PEG copolymers blended with the nanostructured SeO_2_ and BN particles’ nanocomposite matrix resulted in a good improvement in the possibilities of reciprocal influence between the arriving gamma rays and the shielding nanocomposite atoms. We can say that the investigated nanocomposites can be also used as shielding materials against low and high doses of gamma rays.

The $\mu_{L}$ rates tend to decrease as gamma energies increase (as can be seen in Figure 7). It was concluded that the experimental and theoretical $\mu_{L}$ rates showed good harmony and increasing shielding behavior with the change in the type of polymer used to develop gamma-ray-absorbing nanomaterials. Moreover, we also found that the gamma-ray protection features of the nanocomposites improved when the amounts of nanostructured SeO_2_ and BN particles in the nanomaterials were altered.

The $\mu_{L}$ values of our nanocomposites decreased as the gamma-ray energy increased. In the range from 121.788 keV to 1408.006 keV of the ^152^Eu source, when we viewed the sequence of $\mu_{L}$ values for the PS-PEG (1000), PS-PEG (1500), and PS-PEG (10,000) copolymers blended with the nanostructured SeO_2_ nanocomposites, the following results were concluded (Table S2, available in the Supplementary Materials): The experimental $\mu_{L}$ value of the PSNC10 composite, which dissolves 46.2 wt% PS-PEG (1500) copolymer at 121.782 keV, is 0.268; the $\mu_{L}$ of the PSNC6 copolymer is 0.282. In other studied energy values, the SeO_2_ contribution increased the $\mu_{L}$ coefficient. The experimental $\mu_{L}$ of the PSNC2 composite containing 50 wt% SeO_2_ and 50 wt% PSNC6 at 344.279 keV is 0.154. The $\mu_{L}$ value of the PSNC6 copolymer is 0.193. At 344.279 keV, the experimental $\mu_{L}$ values for other compounds increased with the addition of nanostructured SeO_2_ to the polymer.

At 778.904 keV, the experimental $\mu_{L}$ values of the PSNC7, PSNC8, and PSNC15 nanocomposites were lower than the $\mu_{L}$ values of the polymers. This increased the radiation absorption properties of the composites. While the experimental $\mu_{L}$ value of the PSNC6 copolymer containing PS-PEG (1500) was 0.141, this value decreased to 0.110 in the PSNC8 composite containing 30 wt% PS-b-PEG (1500) and 70 wt% SeO_2_. Similarly, the $\mu_{L}$ value of the PSNC7 composite containing 50 wt% PSNC6 and 50 wt% SeO_2_ was found to be 0.131, while the $\mu_{L}$ value of the PSNC10 composite was found to be 0.116. In the PSNC10 composite, the $\mu_{L}$ value of the PS-PEG (1500) copolymer was below the $\mu_{L}$ value of the polymer. At 964.079 keV, the $\mu_{L}$ values of the PSNC8, PSNC9, and PSNC10 composites were 0.107, 0.089, and 0.098, respectively. The $\mu_{L}$ value of the PSNC6 copolymer in composites was 0.111. At 1085.869, 1112.074, and 1408.006 keV, 30 wt%, 70 wt%, 90 wt%, and 53.8 wt% SeO_2_ nanoparticle contributions to polymers caused an increase in $\mu_{L}$ values.

In addition, when we viewed the sequence of $\mu_{L}$ values for the PS-PEG (1000), PS-PEG (1500), and PS-PEG (10,000) copolymers blended with the nanostructured SeO_2_ and BN nanocomposites, the following results were concluded (Table S3, available in the Supplementary Materials): At 121.782 keV, the $\mu_{L}$ values of the PBSNC5 nanocomposite containing 50 wt% PSNC6 and 50 wt% BN, and of the BPSNC9 nanocomposite containing 50 wt% PSNC11 and 50 wt% BN, were calculated as 0.260 and 0.186, respectively. The $\mu_{L}$ values of PSNC6 and PSNC11 copolymers were 0.282 and 0.195, respectively. $\mu_{L}$ values increased when SeO_2_ and BN particles were added to the polymer at 121.782 keV. At 344.279 keV, the $\mu_{L}$ values of PBSNC1 and PBSNC9 composites with 50 wt% BN and 50 wt% copolymer were found to be lower than the $\mu_{L}$ values of their respective polymers. The $\mu_{L}$ value of the PBSNC7 composite containing 5 wt% PSNC6, 5 wt% BN, and 90 wt% SeO_2_ was 0.179, which is lower than the $\mu_{L}$ value of its copolymer (PSNC6’s $\mu_{L}$ is 0.193). The $\mu_{L}$ value of the PBSNC8 composite containing 26.1 wt% polymer, 13 wt% BN, and 60.9 wt% SeO_2_ was 0.169. The $\mu_{L}$ value of the PSNC6 copolymer in the composite decreased with the contribution of 13 wt% BN and 60.9 wt% SeO_2_. At 778.904 keV, the m_L_ value of the 50 wt% BN and 50 wt% PSNC6-doped PBSNC5 composite was lower than the $\mu_{L}$ value of the polymer—the $\mu_{L}$ value of the PBSNC5 nanocomposite was 0.134, while the $\mu_{L}$ value of the polymer is 0.141. A slight increase was observed in the values of other composites containing polymer, BN, and SeO_2_ nanostructured particles in the same energy range.

At 964.079 keV, the $\mu_{L}$ of the PBSNC1 nanocomposite was lower than the m_L_ of its polymer. The $\mu_{L}$ of the PBSNC7 nanocomposite was 0.094. The $\mu_{L}$ of PSNC6 copolymer was 0.111. The $\mu_{L}$ values for polymer composites containing nanostructured SeO_2_ and BN particles at 964.079 keV were slightly higher than the $\mu_{L}$ values of the copolymers. At 1085.869 keV, the $\mu_{L}$ values of the polymers increased slightly to moderately with the contribution of nanostructured SeO_2_ and BN particles. When only 50 wt% nanostructured BN was added to the PSNC1 copolymer, the $\mu_{L}$ value of the copolymer decreased from 0.094 to 0.074, while the $\mu_{L}$ values of other composites increased. The $\mu_{L}$ value of the PBSNC1 nanocomposite obtained by adding 50 wt% nanostructured BN to the 50 wt% PSNC1 copolymer at 1112.074 keV was lower than the $\mu_{L}$ value of the copolymer. The $\mu_{L}$ values of the PBSNC1 nanocomposite and the PSNC1 copolymer were 0.058 and 0.082, respectively. At 1408.006 keV, the $\mu_{L}$ values of the PBSNC1, PBSNC3, PBSNC7, and PBSNC11 nanocomposites, which had lower $\mu_{L}$ values than their polymers, were 0.038, 0.063, 0.041, and 0.073, respectively. It was observed that there were also slight increases in the $\mu_{L}$ values of the other composites. As can be seen from all of our experimental results, the best linear absorption result is obtained when the nanoparticle additive ratio is increased.

A good harmony was observed between the theoretical and experimental $\mu_{m}$ values of our nanocomposites. When we observed the mass attenuation characteristics, the success in shielding against gamma rays was obvious for the PS-b-PEG copolymers blended with both the SeO_2_ and BN nanocomposites, as shown in Figure 8 and Tables S4 and S5 (the tables are present in the Supplementary Materials).

The $\mu_{m}$ values of our nanocomposites reduced as the gamma-ray energy increased. In the range from 121.788 keV to 1408.006 keV of the ^152^Eu source, when we viewed the sequence of $\mu_{mass}$ values for the PS-PEG (1000), PS-PEG (1500), and PS-PEG (10,000) copolymers blended with the nanostructured SeO_2_ nanocomposites, the following results could be concluded (Table S4, present in the Supplementary Materials): The $\mu_{m}$ values of the composites (such as PSNC3, PSNC8, PSNC9, and PSNC10) containing different ratios of polymer and SeO_2_ nanoparticles examined at 121.782 keV were decreased. In general, although the $\mu_{m}$ values of the PSNC12, PSNC13, PSNC14, and PSNC15 nanocomposites containing PSNC11 copolymer were higher than the values of their polymers, the $\mu_{m}$ values of the composites containing 50 wt%, 90 wt%, and 53.8 wt% nanostructured SeO_2_ particles in the PSNC2, PSNC7, PSNC4, and PSNC5 composites containing PSNC1 and PSNC6 copolymers were higher, and it was found that the polymers increased slightly from the $\mu_{m}$ values. It was also found that the $\mu_{m}$ values of the PSNC13, PSNC14, and PSNC15 nanocomposites with only 70 wt%, 90 wt%, and 53.8 wt% nanostructured SeO_2_ particles, respectively, increased at 344.279 keV. At this energy, the $\mu_{m}$ values of the other nanocomposites were lower than the $\mu_{m}$ values of the polymers. The $\mu_{m}$ values for the PSNC2 nanocomposite containing 50 wt% PSNC1 copolymer and 50 wt% SeO_2_, the PSNC7 nanocomposite containing 50 wt% PSNC6 copolymer and 50 wt% SeO_2_, and the PSNC12 nanocomposite containing 50 wt% PSNC11 copolymer and 50 wt% SeO_2_ were 0.092, 0.142, and 0.118, respectively. The $\mu_{m}$ values of the PSNC3 nanocomposite containing 30 wt% PSNC1 copolymer and 70 wt% SeO_2_, and the PSNC8 nanocomposite containing 30 wt% PSNC6 copolymer and 70 wt% SeO_2_, were found to be 0.124 and 0.155, respectively. The $\mu_{m}$ values of the PSNC4 nanocomposite containing 10 wt% PSNC1 copolymer and 90 wt% SeO_2_, and the PSNC9 nanocomposite containing 10 wt% PSNC6 copolymer and 90 wt% SeO_2_, were 0.107 and 0.180 respectively. The $\mu_{m}$ values of the PSNC5 nanocomposite containing 46.2 wt% PSNC1 copolymer and 53.8 wt% SeO_2_ nanoparticles were 0.140 and 0.178; these values are lower than the copolymers’ values. In other words, successful targeted absorption was achieved in the samples at this energy value.

When the $\mu_{m}$ values of nanocomposites are compared with the $\mu_{m}$ values of copolymers at 788.904 keV, it can be seen that radiation absorption success is achieved (Table S4). Of the nanocomposites with 50 wt%, 70 wt%, and 53.8 wt% SeO_2_ nanoparticles, the $\mu_{m}$ values of the PSNC12, PSNC13, and PSNC15 nanocomposites showed an improvement from the $\mu_{m}$ values of their copolymers. At 964.079 keV, the $\mu_{m}$ values of the PSNC2, PSNC12, PSNC13 nanocomposites increased compared to the values of their pure copolymers. The $\mu_{m}$ values of the PSNC7 and PSNC12 nanocomposites blended with 50 wt% SeO_2_ nanoparticles were 0.055 and 0.107, respectively. The PSNC3, PSNC8, and PSNC13; PSNC4, PSNC9, and PSNC14; and PSNC5, PSNC10, and PSNC15 nanocomposites blended with 70 wt%, 90 wt%, and 53.8 wt% SeO_2_ nanostructured particles, respectively, had $\mu_{m}$ values of 0.045, 0.070, and 0.089; 0.088, 0.058, and 0.062; and 0.071, 0.083, and 0.061, respectively. Again, the measurements taken at this energy value maintained good harmony with the theoretical values.

At 1085.869 keV, the $\mu_{m}$ values of the PSNC2, PSNC7, PSNC3, PSNC4, and PSNC5 nanocomposites were lower than the $\mu_{m}$ values of their copolymers; the $\mu_{m}$ values for these nanostructured samples were 0.077, 0.052, 0.071, 0.073, 0.081, and 0.072, respectively. At 1112.074 keV, the $\mu_{m}$ values of some nanocomposites increased or decreased slightly, but results consistent with the expected theoretical values were also obtained at this energy value. The $\mu_{m}$ values of the PSNC7, PSNC3, PSNC8, PSNC4, and PSNC5 nanocomposites containing 50 wt%, 70 wt%, 90 wt%, and 53.8 wt% nanostructured SeO2 particles were found to be 0.041, 0.067, 0.053, 0.072, and 0.075, respectively. At 1408.006 keV, the $\mu_{m}$ values of the PSNC2, PSNC7, PSNC3, PSNC8, PSNC4, PSNC9, and PSNC5 nanocomposites were 0.062, 0.042, 0.047, 0.043, 0.060, 0.043, and 0.045, respectively. In this energy region, the theoretical values were compatible, and the predicted radiation attenuation performance was achieved.

When the composites containing copolymer, SeO_2_, and BN nanoparticles at 121.782 keV were examined, the $\mu_{m}$ values of the PBSNC5, PBSNC9, PBSNC6, PBSNC8, and PBSNC12 nanocomposites were lower than their copolymers’ $\mu_{m}$ values, at 0.151, 0.142, 0.242, 0.263, and 0.329, respectively. At this energy, with the addition of 50 wt% nanostructured BN to the PSNC6 and PSNC11 copolymers, the $\mu_{m}$ values of the PSNC6 and PSNC11 copolymers decreased from 0.283 to 0.151 and from 0.177 to 0.142, respectively. The $\mu_{m}$ value of the PBSNC6 nanocomposite containing 15% PSNC6 + 15% BN + 70% SeO_2_ decreased from 0.283 to 0.242. It was observed that the radiation protection properties increased in the PBSNC3, PBSNC7, and PBSNC11 nanocomposites containing 5 wt% copolymer + 5 wt% BN nanoparticles + 90 wt% SeO_2_ nanoparticles, and in the 344.279, 778.904, 964.079, 1085.869, 1112.074, and 1408.006 keV regions it was seen that the nanocomposites showed the appropriate absorption curves—with some exceptions—compatible with their theoretical values. As a result, it was observed that when the crosslinker in PS-PEG copolymer is PEG-DM (10,000), its radiation absorption property is slightly lower than that of other copolymers, due to its high molecular weight.

The $\mu_{m}$ values tend to decrease as the gamma energy increases (as can be seen in Figure 8). It was concluded that the experimental and theoretical $\mu_{m}$ rates showed good harmony; that is, the PS-b-PEG copolymers blended with nanostructured SeO_2_ and BN particles in a nanocomposite matrix showed good attenuation and protective results against gamma irradiation. It can be concluded that our nanocomposites can also be used as shielding materials against both low and high gamma doses in many areas.

As has been comprehensively underlined for all of our nanocomposites’ results, the experimental and theoretical values demonstrate changes with the change in the type of copolymer used to develop the nanomaterial for the attenuation of and protection against the effects of gamma rays. Furthermore, it was noted that the radiation shielding ability of our nanocomposites increased when the nanostructural proportions of SeO_2_ or BN particles in the nanocomposites were greater. When the $\mu_{L}$ and $\mu_{m}$ values of all of the nanocomposites are perused, it can be seen that the nanostructured composites cultivated in this work display significant and dependable results in terms of radiation absorption and protection.

While the $\mu_{L}$ values of the PS-b-PEG copolymers blended with the nanostructured PbO particles change from 0.397 cm^−1^ to 0.075 cm^−1^, the $\mu_{L}$ values of the PS-b-PEG (1000) copolymer blended with the nanostructured SeO_2_ particles change from 0.557 cm^−1^ to 0.103 cm^−1^ (for 50% PS-b-PEG (1000) copolymer and 50% nanostructured particles) [12]. Additionally, the $\mu_{L}$ values of the PS-b-PEG copolymers blended with the nanostructured PbO and BN particles change from 0.717 cm^−1^ to 0.094 cm^−1^ (for 15% PS-b-PEG (10,000) copolymer, 15% nanostructured BN, and 70% PbO particles) [12], while the $\mu_{L}$ values of the PS-b-PEG (10,000) copolymer blended with the nanostructured SeO_2_ and BN particles change from 0.823 cm^−1^ to 0.096 cm^−1^ (for 15% PS-b-PEG (10,000) copolymer, 15% nanostructured BN, and 70% SeO_2_ particles). Furthermore, the $\mu_{m}$ values of the PS-b-PEG copolymers blended with the nanostructured PbO particles change from 0.261 cm^2^/g to 0.049 cm^2^/g, while the $\mu_{m}$ values of the PS-b-PEG (1000) copolymer blended with the nanostructured SeO_2_ particles change from 0.333 cm^2^/g to 0.062 cm^2^/g (for 50% PS-b-PEG (1000) copolymer and 50% nanostructured particles) [12]. In addition, the $\mu_{m}$ values of the PS-b-PEG copolymers blended with the nanostructured PbO and BN particles change from 0.312 cm^2^/g to 0.041 cm^2^/g (for 15% PS-b-PEG (10,000) copolymer, 15% nanostructured BN, and 70% PbO particles) [12], and the $\mu_{m}$ values of the PS-b-PEG (10,000) copolymer blended with the nanostructured SeO_2_ and BN particles change from 0.329 cm^2^/g to 0.039 cm^2^/g (for 15% PS-b-PEG (10,000) copolymer, 15% nanostructured BN, and 70% SeO_2_ particles).

According to these polymer-based results, nanostructured SeO_2_ particles, as compared to nanostructured lead particles, are an efficient and high-value-added alternative for use in radiation shielding materials. We concluded that the gamma photons and the nanocomposite influence one another; that is, the developed nanocomposites attenuate the gamma rays, which could not penetrate our polymer-based nanomaterial. When the energy was increased, the gamma rays passed through the sample slightly more easily, illustrating that the $\mu_{L}$ and $\mu_{m}$ values were comparatively low in the high-energy gamma rays. According to the results of the nanocomposites we developed, nanocomposites with higher polymer content are more flexible and durable, while nanocomposites with a higher nanometal additive ratio have better gamma attenuation properties.

#### 3.3.2. Half-Value Layer (HVL), Tenth Value Layer (TVL), Mean Free Path (MFP), and Radiation Protection Efficiency (RPE)

The calculated HVL, TVL, MFP, and RPE rates of our nanocomposites are the most influential factors of the gamma shielding effectiveness. The lower the HVL, TVL, and MFP rates, the more influential the properties of the gamma-ray shield. Furthermore, by calculating the RPE rates, the performance of the polymer-based nanocomposites prepared to develop the shield for protection against gamma rays in a wide energy range can be observed. All of these important radiation characteristics were calculated using the equations in Table 2.

**Figure 9:**
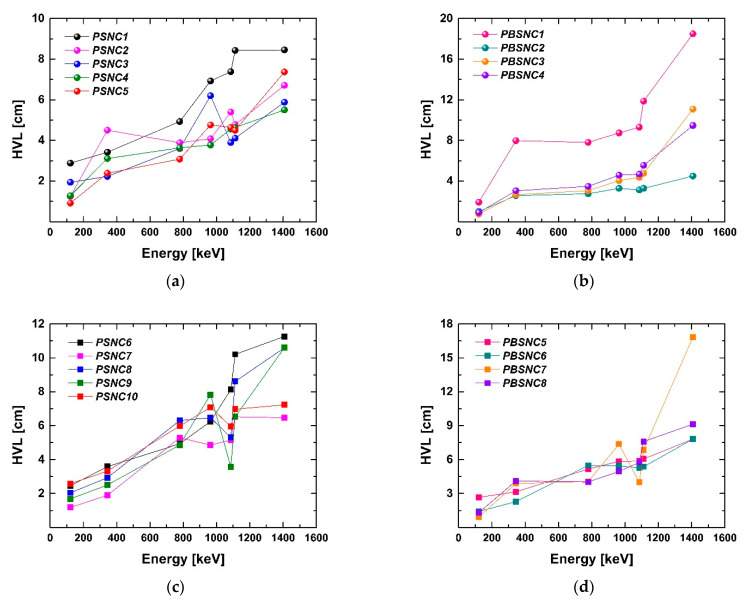

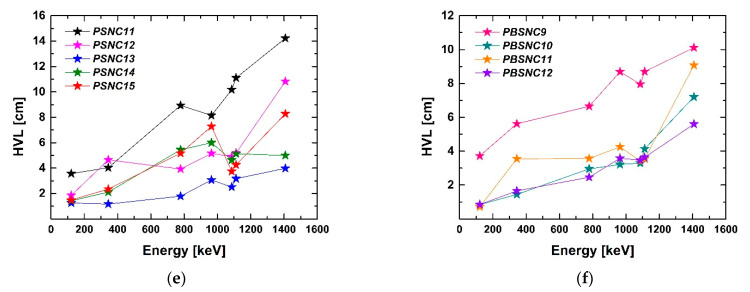
The HVL values of the PS-b-PEG copolymers blended with the nanostructured SeO_2_ and BN particles under a wide range of gamma irradiation energies: PSNC1, PSNC2, PSNC3, PSNC4, and PSNC5 (**a**); PBSNC1, PBSNC2, PBSNC3, and PBSNC4 (**b**); PSNC6, PSNC7, PSNC8, PSNC9, and PSNC10 (**c**); PBSNC5, PBSNC6, PBSNC7, and PBSNC8 (**d**); PSNC11, PSNC12, PSNC13, PSNC14, and PSNC15 (**e**); PBSNC9, PBSNC10, PBSNC11, and PBSNC12 (**f**).

**Figure 10:**
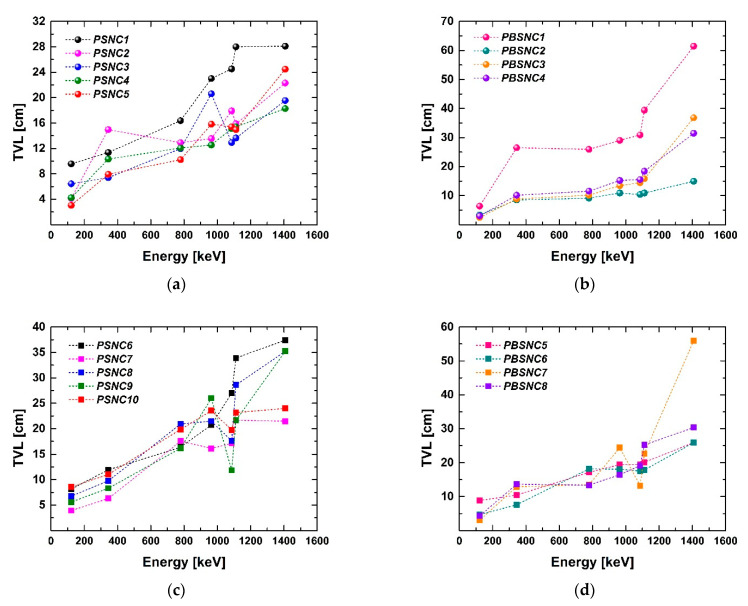

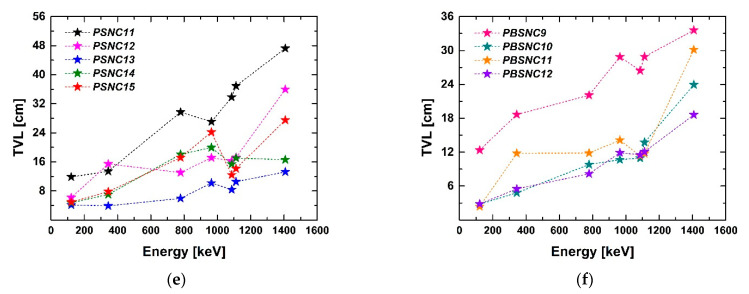
The TVL values of the PS-b-PEG copolymers blended with the nanostructured SeO_2_ and BN particles under a wide range of gamma irradiation energies: PSNC1, PSNC2, PSNC3, PSNC4, and PSNC5 (**a**); PBSNC1, PBSNC2, PBSNC3, and PBSNC4 (**b**); PSNC6, PSNC7, PSNC8, PSNC9, and PSNC10 (**c**); PBSNC5, PBSNC6, PBSNC7, and PBSNC8 (**d**); PSNC11, PSNC12, PSNC13, PSNC14, and PSNC15 (**e**); PBSNC9, PBSNC10, PBSNC11, and PBSNC12 (**f**).

As can be seen in Figure 9 and Figure 10 and Tables S6 and S7 (the tables are present in the Supplementary Materials), the HVL and TVL rates show the thickness required to stop half of the gamma rays or radiation. The HVL and TVL rates tend to increase steadily in the range from 121.788 keV to 1408.006 keV. It was concluded that the HVL and TVL rates showed good harmony with one another, and increasing shielding behavior with the change in the type of polymer used to develop gamma-ray-absorbing nanomaterials. Furthermore, it was also found that the gamma irradiation protection attributes of the nanocomposites increased when the amounts of nanostructured SeO_2_ and BN particles in the nanomaterials were increased.

The HVL rates are a crucial indicator of the shielding ability; that is, it can be said that the smaller the HVL rate of the material, the better the radiation attenuation. Thus, the HVL rates of our nanocomposites increase as the gamma-ray energy increases. In the range from 121.788 keV to 1408.006 keV of the ^152^Eu source, when we viewed the sequence of HVL values for the PS-PEG (1000), PS-PEG (1500), and PS-PEG (10,000) copolymers blended with the nanostructured SeO_2_ nanocomposites, the following results could be concluded (Table S6, present in the Supplementary Materials): PSNC4 < PSNC3 < PSNC2 < PSNC5 < PSNC1, PSNC7 < PSNC10 < PSNC8 < PSNC9 < PSNC6, and PSNC13 < PSNC14 < PSNC15 < PSNC12 < PSNC11.

In addition, when we viewed the sequence of HVL values for the PS-PEG (1000), PS-PEG (1500), and PS-PEG (10,000) copolymers blended with the nanostructured SeO_2_ and BN nanocomposites, the following results could be concluded (Table S7, present in the Supplementary Materials): PBSNC2 < PBSNC4 < PBSNC3 < PBSNC1, PBSNC5 < PBSNC6 < PBSNC8 < PBSNC7, and PBSNC12< PBSNC10 < PBSNC11 < PBSNC9. Furthermore, we compared the sequence of HVL rates for the PS-PEG (1000), PS-PEG (1500), and PS-PEG (10,000) copolymers non-blended with the nanostructured particles in Table 1, concluding that PSNC1 < PSNC6 < PSNC11.

While the HVL rates of the PS-b-PEG copolymers blended with the nanostructured PbO particles changed from 1.746 cm to 9.249 cm, the HVL rates of the PS-b-PEG (1000) copolymer blended with the nanostructured SeO_2_ particles changed from 1.245 cm to 6.713 cm, the PS-b-PEG copolymers blended with the nanostructured PbO particles changed from 1.407 cm to 11.007 cm, and the HVL rates of the PS-b-PEG (1500) copolymer blended with the nanostructured SeO_2_ particles changed from 1.178 cm to 6.466 cm (for 50% PS-b-PEG copolymer and 50% nanostructured particles) [12]. Furthermore, the HVL rates of the PS-b-PEG copolymers blended with the nanostructured PbO and BN particles changed from 0.967 cm to 7.347 cm (for 15% PS-b-PEG (10,000) copolymer, 15% nanostructured BN, and 70% PbO particles) [12], and the HVL values of the PS-b-PEG (10,000) copolymer blended with the nanostructured SeO_2_ and BN particles changed from 0.843 cm to 7.203 cm (for 15% PS-b-PEG (10,000) copolymer, 15% nanostructured BN and 70% SeO_2_ particles). Specifically, the nanostructured SeO_2_ additive reduced the thickness by increasing the radiation absorption efficiency.

The TVL rates are a crucial indicator of the shielding ability; that is, it can be said that the lower the TVL rate of the material, the better the radiation shielding performance. Thus, the TVL rates of our nanocomposites increases as the gamma-ray energy increased. In the interval from 121.788 keV to 1408.006 keV of the ^152^Eu source, when we viewed the sequence of TVL values for the PS-PEG (1000), PS-PEG (1500), and PS-PEG (10,000) copolymers blended with the nanostructured SeO_2_ nanocomposites, the following results could be concluded (Table S6, present in the Supplementary Materials): PSNC4 < PSNC3 < PSNC2 < PSNC5 < PSNC1, PSNC7 < PSNC10 < PSNC8 < PSNC9 < PSNC6, and PSNC13 < PSNC14 < PSNC15 < PSNC12 < PSNC11.

In addition, when we viewed the sequence of TVL values for the PS-PEG (1000), PS-PEG (1500), and PS-PEG (10,000) copolymers blended with the nanostructured SeO_2_ and BN nanocomposites, the following results could be concluded (Table S7, present in the Supplementary Materials): PBSNC2 < PBSNC4 < PBSNC3 < PBSNC1, PBSNC5 < PBSNC6 < PBSNC8 < PBSNC7, and PBSNC12 < PBSNC10 < PBSNC11 < PBSNC9. Furthermore, we compared the sequence of TVL rates for the PS-PEG (1000), PS-PEG (1500), and PS-PEG (10,000) copolymers non-blended with the nanostructured particles in Table 1, concluding that PSNC1 < PSNC6 < PSNC11.

While the TVL rates of the PS-b-PEG copolymers blended with the nanostructured PbO particles changed from 5.799 cm to 30.725 cm, the TVL rates of the PS-b-PEG (1000) copolymer blended with the nanostructured SeO_2_ particles changed from 4.137 cm to 22.299 cm, the PS-b-PEG copolymers blended with the nanostructured PbO particles changed from 4.673 cm to 36.564 cm, and the TVL rates of the PS-b-PEG (1500) copolymer blended with the nanostructured SeO_2_ particles changed from 3.913 cm to 21.478 cm (for 50% PS-b-PEG copolymer and 50% nanostructured particle) [12]. Furthermore, the TVL rates of the PS-b-PEG copolymers blended with the nanostructured PbO and BN particles changed from 3.213 cm to 24.405 cm (for 15% PS-b-PEG (10,000) copolymer, 15% nanostructured BN, and 70% PbO particles) [12], and the TVL values of the PS-b-PEG (10,000) copolymer blended with the nanostructured SeO_2_ and BN particles changed from 2.799 cm to 23.929 cm (for 15% PS-b-PEG (10,000) copolymer, 15% nanostructured BN and 70% SeO_2_ particles). Specifically, the nanostructured SeO_2_ additive reduced the thickness by increasing the radiation absorption efficiency.

**Figure 11:**
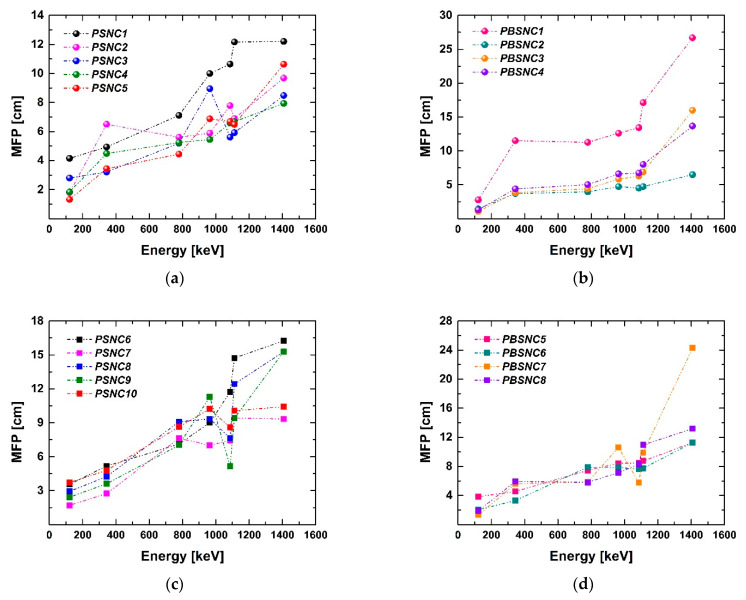

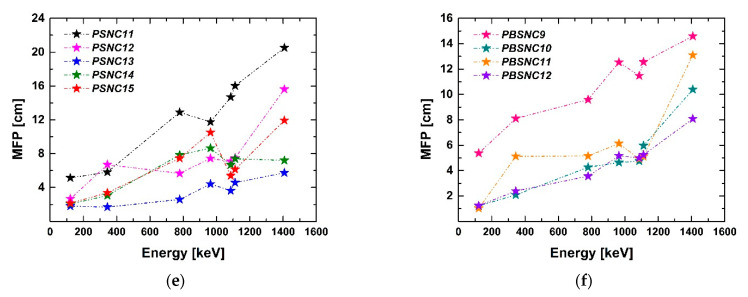
The MFP rates of the PS-b-PEG copolymers blended with the nanostructured SeO_2_ and BN particles under a wide range of gamma irradiation energies: PSNC1, PSNC2, PSNC3, PSNC4, and PSNC5 (**a**); PBSNC1, PBSNC2, PBSNC3, and PBSNC4 (**b**); PSNC6, PSNC7, PSNC8, PSNC9, and PSNC10 (**c**); PBSNC5, PBSNC6, PBSNC7, and PBSNC8 (**d**); PSNC11, PSNC12, PSNC13, PSNC14, and PSNC15 (**e**); PBSNC9, PBSNC10, PBSNC11, and PBSNC12 (**f**).

The MFP value is the average path on which gamma photons travel before an interaction takes place (between consecutive coactions), as shown in Figure 11. We viewed the sequence of MFP values for the PS-PEG (1000), PS-PEG (1500), and PS-PEG (10,000) copolymers blended with the nanostructured SeO_2_ nanocomposites; the following results can be concluded (Table S8, present in the Supplementary Materials): PSNC4 < PSNC3 < PSNC2 < PSNC5 < PSNC1, PSNC7 < PSNC10 < PSNC8 < PSNC9 < PSNC6, and PSNC13 < PSNC14 < PSNC15 < PSNC12 < PSNC11.

In addition, when we viewed the sequence of MFP values for the PS-PEG (1000), PS-PEG (1500), and PS-PEG (10,000) copolymers blended with the nanostructured SeO_2_ and BN nanocomposites, the following results could be concluded (Table S9 present in the Supplementary Materials): PBSNC2 < PBSNC4 < PBSNC3 < PBSNC1, PBSNC5 < PBSNC6 < PBSNC8 < PBSNC7, and PBSNC12 < PBSNC10 < PBSNC11 < PBSNC9. Furthermore, we compared the sequence of MFP rates for the PS-PEG (1000), PS-PEG (1500), and PS-PEG (10,000) copolymers non-blended with the nanostructured particles in Table 1, concluding that PSNC1 < PSNC6 < PSNC11.

While the MFP rates of the PS-b-PEG copolymers blended with the nanostructured PbO particles changed from 2.518 cm to 13.344 cm, the MFP rates of the PS-b-PEG (1000) copolymer blended with the nanostructured SeO_2_ particles changed from 1.797 cm to 9.685 cm, the PS-b-PEG copolymers blended with the nanostructured PbO particles changed from 2.029 cm to 15.879 cm, and the MFP rates of the PS-b-PEG (1500) copolymer blended with the nanostructured SeO_2_ particles changes from 1.699 cm to 9.328 cm (for 50% PS-b-PEG copolymer and 50% nanostructured particles) [12]. Furthermore, the MFP rates of the PS-b-PEG copolymers blended with the nanostructured PbO and BN particles changed from 1.395 cm to 10.599 cm (for 15% PS-b-PEG (10,000) copolymer, 15% nanostructured BN, and 70% PbO particles) [12], and the MFP values of the PS-b-PEG (10,000) copolymer blended with the nanostructured SeO_2_ and BN particles changed from 1.216 cm to 10.392 cm (for 15% PS-b-PEG (10,000) copolymer, 15% nanostructured BN and 70% SeO_2_ particles). Specifically, the nanostructured SeO_2_ additive reduced the thickness by increasing the radiation absorption efficiency.

The lower the MFP values, the better the shielding efficiency of the nanocomposites. Our conclusions indicate that the MFP values of our nanocomposites increased with the increasing gamma energy values.

**Figure 12:**
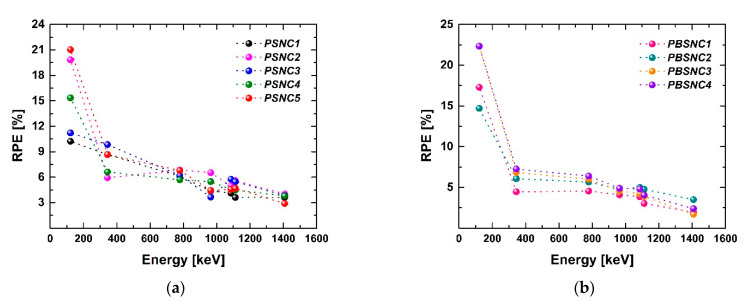

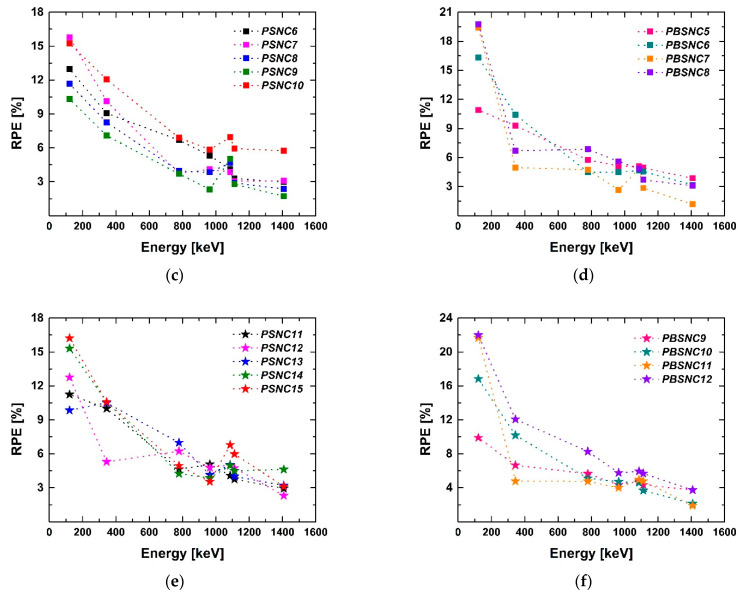
The RPE rates of the PS-b-PEG copolymers blended with the nanostructured SeO_2_ and BN particles under a wide range of gamma irradiation energies: PSNC1, PSNC2, PSNC3, PSNC4, and PSNC5 (**a**); PBSNC1, PBSNC2, PBSNC3, and PBSNC4 (**b**); PSNC6, PSNC7, PSNC8, PSNC9, and PSNC10 (**c**); PBSNC5, PBSNC6, PBSNC7, and PBSNC8 (**d**); PSNC11, PSNC12, PSNC13, PSNC14, and PSNC15 (**e**); PBSNC9, PBSNC10, PBSNC11, and PBSNC12 (**f**).

Furthermore, by computing the RPE rates, the ability of our polymer-based nanocomposites developed to detect the attenuation behaviors of the gamma rays in the wide energy range can be observed. All of our results indicate that our copolymers blended with the nanostructured SeO_2_ and BN nanocomposites show fine shielding performance in the face of gamma rays. Figure 12 and Tables S8 and S9 (the tables are present in the Supplementary Materials) indicate that the highest RPE rates were acquired at 121.7817 keV, and the maximum rate was ~22.334%. When we analyzed all of our PS-b-PEG copolymers blended with the nanostructured SeO_2_ and BN nanocomposites, we deduced that the highest RPE rates and perfect gamma radiation attenuation behavior were attributed to the most abundant ingredient in the nanocomposites. It should also be noted that when the gamma radiation energy increases, the RPE rates decrease, meaning that these decreases in the RPE rates confirm that all of our results are compatible with the other attenuation parameters (i.e., $\mu_{L}$, $\mu_{m}$, HVL, TVL, MFP) computed and experimentally detected. These conclusions obtained are consistent with the results of the research composites developed for gamma-ray shielding with various nanostructured additives, including polymers [11,12,30,31,32,33]. As a result, the current alterations in nanoparticle proportions are quite efficacious for diminishing the influence of gamma radiation.

**Figure 13:**
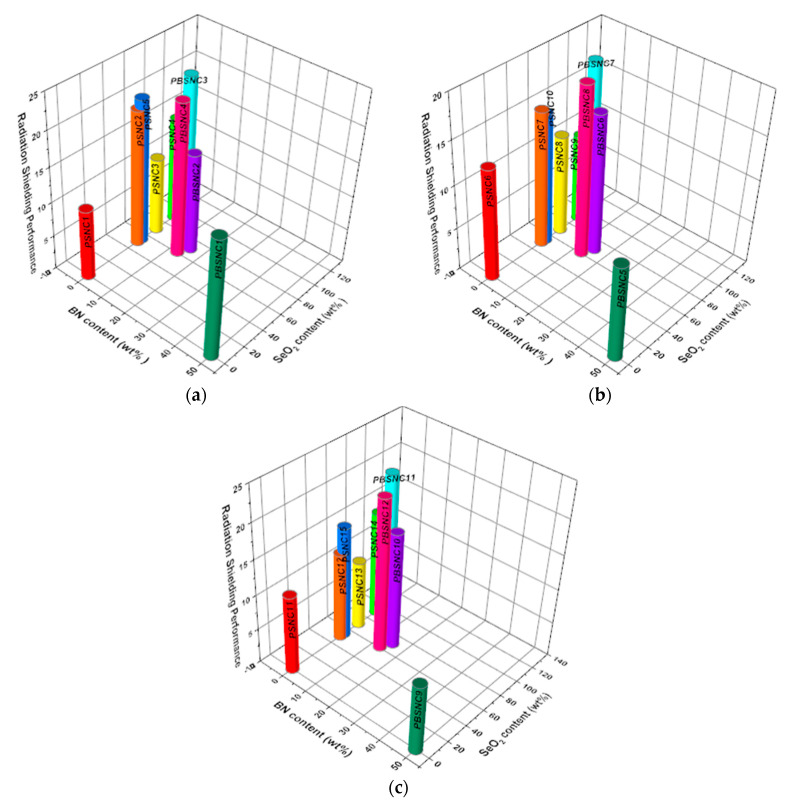
The radiation shielding performance of the PS-b-PEG copolymers blended with the nanostructured SeO_2_ and BN particles under a wide range of gamma irradiation energies: PS-PEG (1000) (**a**), PS-PEG (1500) (**b**), and PS-PEG (10,000) (**c**) copolymer and nanocomposite groups.

The graphs (in Figure 13) show the radiation shielding performance of the PS-PEG (1000)-SeO_2_-BN, PS-PEG (1500)-SeO_2_-BN, and PS-PEG (10,000)-SeO_2_-BN nanocomposites, respectively. In Figure 13a, it can be seen that when 50 wt% SeO_2_ particles were added to the PSNC1 copolymer, the radiation shielding performance value of the PSNC2 nanocomposite increased to 19.824. When we increased the SeO_2_ ratio to 53 wt%, PSNC5 nanocomposite was the nanocomposite with the best radiation shielding performance value of the SeO_2_-doped nanocomposites in the PS-PEG (1000) copolymer group, with a value of 21.029. When PS-PEG (1000)-SeO_2_-BN-doped nanocomposites are examined, the best radiation shielding performance value in this set is 22.334 in the PBSNC3 and PBSNC4 nanocomposites containing PS-PEG (1000) 5 wt%-SeO_2_ 90 wt%-BN 5 wt% and PS-PEG (1000) 26.1 wt%-SeO_2_ 60.9 wt%-BN 13 wt%.

When 50 wt% SeO_2_ was added to the PSNC6 copolymer in the PS-PEG (1500) set containing only SeO_2_, the radiation shielding performance value in the PSNC7 nanocomposite increased to 15.787, and the best radiation shielding performance value in the set was reached, as shown in Figure 13b. When 13 wt% BN nanoparticles were added to the nanocomposite, the best radiation shielding performance value was obtained with PBSNC8, which consists of PS-PEG (1500) 26.1 wt%-SeO_2_ 60.9 wt%-BN 13 wt%. The radiation shielding performance values for PS-PEG (10,000) are presented in Figure 13c. The radiation shielding performance value of the PSNC11 polymer increased from 11.240 to 12.760 with the addition of 50 wt% SeO_2_. The radiation shielding performance value of the PSNC15 nanocomposite, in which the SeO_2_ ratio was increased to 53.8 wt%, reached the best value in the set, with a value of 16.222. When the PS-PEG (10,000)-SeO_2_-BN-doped group is examined, it can be seen that the best shielding performance values were 21.668 and 21.986, in the PBSNC11 and PBSNC12 nanocomposites with PS-PEG (10,000) 5 wt%- SeO_2_ 90 wt%-BN 5 wt% and PS-PEG (10,000) 26.1 wt%-SeO_2_ 60.9 wt%-BN 13 wt%, respectively.

## 4. Conclusions

By detecting the outputs of nanostructured SeO_2_ and BN particle blended/unblended PS-b-PEG-based composite tablets with the experimental system, the infrastructure of which was created with theoretical calculations via the radiation parameters, the shielding test properties of these composites were comprehensively obtained with the ^152^Eu gamma radioisotope source. Furthermore, morphological and temperature degradation characteristics also were measured.

As a result of the detection, observation, scrutiny, and comparative assessment of the detected and computed values, it can be concluded that the radiation shielding factors obtained from the irradiation outputs and the radiation shielding factors obtained from the theoretical computations are quite harmonious with one another. It can be said that the negligible differences mainly depend on the distributions in the materials and the laboratory conditions. It was noted that the addition of nanostructured SeO_2_ and BN particles to PS-b-PEG copolymer-structured composite materials increases the radiation shielding and protection against harmful gamma rays provided by the materials. In this context, the behavior of SeO_2_ and BN blended/unblended nanocomposites against a gamma radioisotope source with a wide energy range was examined, and an application-oriented study that can be used in areas such as nuclear technology was carried out, to the results of which can contribute to the scientific literature. It was observed that when 50 wt% nanostructured SeO_2_ was added to the PSNC1 copolymer, the radiation shielding performance of the PSNC2 nanocomposite increased. When we increased the SeO_2_ ratio to 53 wt%, PSNC5 nanocomposite was the nanocomposite with the best radiation shielding performance value of the nanostructured SeO_2_-blended nanocomposites in the PS-PEG (1000) copolymer group. When the nanostructured SeO_2_ and BN blended with PS-PEG (1000) copolymer nanocomposites were examined, the best radiation shielding performance rates in this set were obtained in the PBSNC3 and PBSNC4 nanocomposites. When 50 wt% nanostructured SeO_2_ was added to the PSNC6 copolymer, the radiation shielding performance value in the PSNC7 nanocomposite increased, and the best radiation shielding performance value in the set was reached. When 13 wt% nanostructured BN was added to the nanocomposite, the best radiation shielding performance value was obtained with the PBSNC8 sample. The radiation shielding performance of the PSNC11 copolymer increased with the addition of 50 wt% nanostructured SeO_2_. The radiation shielding performance value of the PSNC15 nanocomposite, in which the SeO_2_ ratio was increased to 53.8 wt%, was the best value in the set. When the nanostructured SeO_2_-BN blended nanocomposites in the PS-PEG (10,000) copolymer group are examined, it can be seen that the best shielding performance values were attained in the PBSNC11 and PBSNC12 nanocomposites.

The TGA curves of our nanocomposites were exhaustively analyzed in order to characterize the decomposition of the PS-b-PEG copolymers blended with the nanostructured SeO_2_ and BN particles. The nanocomposites with a higher wt% of nanostructured SeO_2_ and BN particles represented preferable thermal uniformity, and acted like walls protecting the chains formed on the surface from decomposition. Consequently, all thermogram ratios demonstrated that our PS-b-PEG structured nanocomposites were thermally balanced.

The SEM and TEM photographs of the surface morphology of our nanocomposites are a contribution to the literature. Information about the distribution of the SeO_2_ nanoparticles was obtained from EDX graphics. As the molecular weight of the macro-crosslinker increases, the polymer particles become more prominent, and the nanoparticles are more homogeneously dispersed on the surface.

Another important result from the TEM photographs is that when BN nanoparticles are added to the nanocomposite, they noticeably change the distribution and particle structure of SeO_2_ nanoparticles in the composite.

Due to the added value of polymers, such as being cheap, light, flexible, and durable, our nanocomposites, which we have developed and researched with polymer structures and nanometal particles, have good potential for minimizing the harmful effects of radiation and protecting against them.

## Figures and Tables

**Table 1:** Nanocomposite labels and configurations (by weight %) of the advanced nanocomposites.

Line	Nanocomposite Label	PS-b-PEG Type	PS-b-PEG (wt%)	BN (wt%)	SeO_2_ (wt%)
1	PSNC1	1000	100	0	0
2	PSNC2	1000	50	0	50
3	PSNC3	1000	30	0	70
4	PSNC4	1000	10	0	90
5	PSNC5	1000	46.2	0	53.8
6	PSNC6	1500	100	0	0
7	PSNC7	1500	50	0	50
8	PSNC8	1500	30	0	70
9	PSNC9	1500	10	0	90
10	PSNC10	1500	46.2	0	53.8
11	PSNC11	10,000	100	0	0
12	PSNC12	10,000	50	0	50
13	PSNC13	10,000	30	0	70
14	PSNC14	10,000	10	0	90
15	PSNC15	10,000	46.2	0	53.8
16	PBSNC1	1000	50	50	0
17	PBSNC2	1000	15	15	70
18	PBSNC3	1000	5	5	90
19	PBSNC4	1000	26.1	13	60.9
20	PBSNC5	1500	50	50	0
21	PBSNC6	1500	15	15	70
22	PBSNC7	1500	5	5	90
23	PBSNC8	1500	26.1	13	60.9
24	PBSNC9	10,000	50	50	0
25	PBSNC10	10,000	15	15	70
26	PBSNC11	10,000	5	5	90
27	PBSNC12	10,000	26.1	13	60.9

**Table 2:** Calculation equations of radiation shielding factors for the advanced nanocomposites.

Radiation Shielding Factors	Units	Equations	References
$\mathrm{Mass}\;\mathrm{attenuation}\;\mathrm{coefficient}\;(\mu_{m}$)	cm^2^/g	$\mu_{m}=\frac{\mu }{\rho }=\sum w_{i}{\left(\frac{\mu }{\rho }\right)}_{i}$	[11,12]
$\mathrm{Linear}\;\mathrm{attenuation}\;\mathrm{coefficient}\;(\mu_{L}$)	cm^−1^	$I=I_{0}e^{-\mu_{L}x}$	[11,12]
Radiation protection efficiency (RPE)	-	$RPE\left(\%\right)=\left(1-\frac{I}{I_{0}}\right)\times 100$	[11,12]
Half-value layer (HVL)	cm	$HVL=X_{h}=\frac{\ln2}{\mu }\approx \frac{0.693}{\mu }$	[11,12]
Tenth-value layer (TVL)	cm	$TVL=X_{t}=\frac{\ln10}{\mu }\approx \frac{2.303}{\mu }$	[11,12]
Mean free path (MFP)	cm	$MFP=X_{m}=\frac{1}{\mu }$	[11,12]

## Data Availability

The data presented in this study are available on request from the corresponding author.

## Supplementary Materials

The following are available online at https://www.mdpi.com/article/10.3390/nano12030297/s1: Section S.1.: Thermogravimetric analysis (TGA) outcomes of the PS-b-PEG copolymers blended with the nanostructured SeO_2_ and BN particles, and of the pure nanostructured SeO_2_ and BN particles; Table S1: TGA outputs of the PS-b-PEG copolymers blended with the nanostructured SeO_2_ and BN particles, and of the pure nanostructured SeO_2_ and BN particles. Section S.2.: Gamma irradiation results; Section S.2.1.: Linear ($\mu_{L}$) and mass attenuation ($\mu_{m}$) coefficients; Table S2: The $\mu_{L}$ rates of the PS-b-PEG copolymers blended with the nanostructured SeO_2_ particles; Table S3: The $\mu_{L}$ rates of the PS-b-PEG copolymers blended with the nanostructured SeO_2_ and BN particles; Table S4: The $\mu_{m}$ rates of the PS-b-PEG copolymers blended with the nanostructured SeO_2_ particles; Table S5: The $\mu_{m}$ rates of the PS-b-PEG copolymers blended with the nanostructured SeO_2_ and BN particles; Section S.2.2.: Half-value layer (HVL), tenth-value layer (TVL), mean free path (MFP), and radiation protection efficiency (RPE); Table S6: The HVL and TVL rates of the PS-b-PEG copolymers blended with the nanostructured SeO_2_ particles; Table S7: The HVL and TVL rates of the PS-b-PEG copolymers blended with the nanostructured SeO_2_ and BN particles; Table S8: The MFP and RPE rates of the PS-b-PEG copolymers blended with the nanostructured SeO_2_ particles; Table S9: The MFP and RPE rates of the PS-b-PEG copolymers blended with the nanostructured SeO_2_ and BN particles; Section S.3.: Characterization of polymer–nanostructured-particle-based nanocomposites via SEM; Figure S1: SEM images of PSNC1 (a,b) and PSNC4 (c,d) nanocomposites; Figure S2: SEM images of PSNC9 (a,b), PSNC14 (c,d), PBSNC2 (e,f), PBSNC6 (g,h), and PBSNC10 (i,j) nanocomposites.
