# Supplementary material for: Radiation Shielding Tests of Crosslinked Polystyrene-b-Polyethyleneglycol Block Copolymers Blended with Nanostructured Selenium Dioxide and Boron Nitride Particles
Source: Nanomaterials (Basel). 2022 Jan 18;12(3):297. doi: 10.3390/nano12030297 (PMC8837946; doi:10.3390/nano12030297)
Supplement: Supplementary file 1 [file nanomaterials-12-00297-s001.zip › nanomaterials-1526470-supplementary.pdf]

nanomaterials

MDPI

Supplementary Materials
Radiation Shielding Tests of Crosslinked Polystyrene-b-Polyethyleneglycol Block Copolymers Blended with Nanostructured Selenium Dioxide and Boron Nitride Particles

Zehra Merve Cinan [1], Burcu Erol [2], Taylan Baskan [1], Saliha Mutlu [3], Bülend Ortaç [4], Sevil Savaskan Yilmaz [3] and Ahmet Hakan Yilmaz [1,*]

[1] Faculty of Sciences, Department of Physics, Karadeniz Technical University, Trabzon 61080, Turkey; m_cinan@ktu.edu.tr (Z.M.C.); taylanbaskan@ktu.edu.tr (T.B.)
[2] Faculty of Arts and Sciences, Department of Physics, Recep Tayyip Erdoğan University, Rize 53100, Turkey; burcu.karayunus@erdogan.edu.tr
[3] Faculty of Sciences, Department of Chemistry, Karadeniz Technical University, Trabzon 61080, Turkey; salihamutlu@ktu.edu.tr (S.M.); sevily@ktu.edu.tr (S.S.Y.)
[4] National Nanotechnology Research Center and Institute of Materials Science and Nanotechnology, Bilkent University, Ankara 06800, Turkey; ortac@unam.bilkent.edu.tr
* Correspondence: Correspondence: hakany@ktu.edu.tr; Tel.: +90-542-385-9893

S1. Thermogravimetric Analysis (TGA) Outcomes of the PS-b-PEG Copolymers Blended with the Nanostructured SeO_2 and BN Particles, and the Pure Nanostructured SeO_2 and BN Particles

Table S1. TGA outputs of the PS-b-PEG copolymers blended with the nanostructured SeO_2 and BN particles, and the pure nanostructured SeO_2 and BN particles.

PSNC4		PSNC9		PSNC14	
Temperature (°C)	wt (%)	Temperature (°C)	wt (%)	Temperature (°C)	wt (%)
76.2	96.9	73.1	95.1	50.4	97.9
122.6	87.5	122.8	91.5	121.6	88.2
251.4	83.0	203.0	87.0	217.7	82.6
294.3	37.1	246.5	75.3	252.7	66.8
469.0	14.8	322.0	70.7	324.5	58.1
662.0	6.7	419.6	23.7	451.8	9.1
-	-	565.9	18.7	606.6	−0.6
-	-	631.5	8.2	-	-
**PBSNC2**		**PBSNC6**		**PBSNC10**	
**Temperature (°C)**	**wt (%)**	**Temperature (°C)**	**wt (%)**	**Temperature (°C)**	**wt (%)**
45.7	98.4	67.6	96.0	85.0	95.1
82.8	91.2	103.8	89.0	138.2	85.2
203.0	83.7	214.9	84.4	222.0	81.8
277.7	59.6	281.5	47.8	302.2	21.8
452.1	45.0	457.7	15.8	434.1	12.3
658.4	40.8	667.0	14.1	565.2	10.3

SeO_2		BN	
Temperature (°C)	wt (%)	Temperature (°C)	wt (%)

78.3	99.8	45.3	95.1
318.1	99.7	46.0	83.9
516.6	−36.9	84.1	58.0
661.6	−36.7	431.7	57.7
-	-	624.6	57.4

S2. Gamma Irradiation Results

S2.1. Linear (μ_L) and Mass Attenuation (μ_m) Coefficients

Table S2. The μ_L rates of the PS-b-PEG copolymers blended with the nanostructured SeO_2 particles.

NC Label	PSNC1		PSNC2		PSNC3		PSNC4		PSNC5	
	$\mu_L\ (cm^{-1})$									
Energy (keV)	EXP.	THEO.	EXP.	THEO.	EXP.	THEO.	EXP.	THEO.	EXP.	THEO.
121.782	0.241	0.274	0.557	0.462	0.357	0.629	0.543	0.767	0.757	0.786
344.279	0.203	0.193	0.154	0.203	0.311	0.238	0.223	0.254	0.290	0.334
778.904	0.141	0.137	0.178	0.137	0.193	0.158	0.191	0.166	0.225	0.226
964.079	0.100	0.123	0.170	0.124	0.112	0.142	0.184	0.149	0.146	0.204
1085.869	0.094	0.116	0.129	0.117	0.178	0.134	0.152	0.140	0.150	0.192
1112.074	0.082	0.115	0.145	0.115	0.169	0.132	0.150	0.138	0.154	0.189
1408.006	0.082	0.102	0.103	0.102	0.118	0.117	0.126	0.123	0.094	0.168
NC Label	PSNC6		PSNC7		PSNC8		PSNC9		PSNC10	
	$\mu_L\ (cm^{-1})$									
Energy (keV)	EXP.	THEO.	EXP.	THEO.	EXP.	THEO.	EXP.	THEO.	EXP.	THEO.
121.782	0.282	0.250	0.589	0629	0.340	0.574	0.412	0.889	0.268	0.398
344.279	0.193	0.176	0.365	0.276	0.236	0.217	0.277	0.294	0.209	0.169
778.904	0.141	0.124	0.131	0.187	0.110	0.144	0.143	0.192	0.116	0.114
964.079	0.111	0.112	0.143	0.168	0.107	0.130	0.089	0.172	0.098	0.103
1085.869	0.085	0.106	0.135	0.159	0.131	0.122	0.194	0.162	0.117	0.097
1112.074	0.068	0.105	0.106	0.157	0.081	0.121	0.106	0.160	0.099	0.096
1408.006	0.062	0.093	0.107	0.139	0.065	0.107	0.065	0.142	0.096	0.085
NC Label	PSNC11		PSNC12		PSNC13		PSNC14		PSNC15	
	$\mu_L\ (cm^{-1})$									
Energy (keV)	EXP.	THEO.	EXP.	THEO.	EXP.	THEO.	EXP.	THEO.	EXP.	THEO.
121.782	0.195	0.202	0.374	0.503	0.554	1.121	0.487	0.690	0.467	0.647
344.279	0.172	0.142	0.149	0.220	0.593	0.423	0.328	0.228	0.295	0.275
778.904	0.078	0.100	0.177	0.150	0.387	0.282	0.127	0.149	0.134	0.186
964.079	0.085	0.091	0.134	0.135	0.227	0.254	0.116	0.134	0.095	0.168
1085.869	0.068	0.086	0.141	0.127	0.276	0.239	0.150	0.126	0.185	0.158
1112.074	0.062	0.085	0.134	0.125	0.219	0.236	0.135	0.124	0.163	0.156
1408.006	0.049	0.075	0.064	0.111	0.174	0.209	0.139	0.110	0.084	0.138

Table S3. The μ_L rates of the PS-b-PEG copolymers blended with the nanostructured SeO_2 and BN particles.

NC Label	PBSNC1		PBSNC2		PBSNC3		PBSNC4	
	$\mu_L\ (cm^{-1})$							
Energy (keV)	EXP.	THEO.	EXP.	THEO.	EXP.	THEO.	EXP.	THEO.
121.782	0.362	0.213	0.688	0.889	0.929	0.852	0.764	0.674
344.279	0.087	0.150	0.271	0.333	0.261	0.281	0.227	0.269
778.904	0.089	0.106	0.252	0.222	0.227	0.183	0.199	0.181

964.079	0.079	0.096	0.211	0.199	0.172	0.165	0.151	0.163
1085.869	0.074	0.091	0.222	0.188	0.159	0.155	0.148	0.153
1112.074	0.058	0.089	0.211	0.185	0.146	0.153	0.125	0.152
1408.006	0.038	0.079	0.154	0.164	0.063	0.136	0.073	0.134
**NC Label**	**PBSNC5**		**PBSNC6**		**PBSNC7**		**PBSNC8**	
	$\mu_L\ (cm^{-1})$							
**Energy (keV)**	**EXP.**	**THEO.**	**EXP.**	**THEO.**	**EXP.**	**THEO.**	**EXP.**	**THEO.**
121.782	0.260	0.250	0.492	0.565	0.756	0.816	0.534	0.541
344.279	0.220	0.176	0.303	0.212	0.179	0.269	0.169	0.217
778.904	0.134	0.125	0.127	0.141	0.170	0.176	0.172	0.145
964.079	0.119	0.113	0.127	0.127	0.094	0.158	0.140	0.131
1085.869	0.118	0.106	0.131	0.119	0.174	0.148	0.120	0.123
1112.074	0.114	0.105	0.129	0.118	0.101	0.147	0.091	0.122
1408.006	0.089	0.093	0.089	0.104	0.041	0.130	0.076	0.108
**NC Label**	**PBSNC9**		**PBSNC10**		**PBSNC11**		**PBSNC12**	
	$\mu_L\ (cm^{-1})$							
**Energy (keV)**	**EXP.**	**THEO.**	**EXP.**	**THEO.**	**EXP.**	**THEO.**	**EXP.**	**THEO.**
121.782	0.186	0.204	0.823	0.881	0.973	0.901	0.809	0.719
344.279	0.123	0.141	0.479	0.330	0.195	0.297	0.419	0.288
778.904	0.104	0.099	0.235	0.219	0.194	0.194	0.282	0.193
964.079	0.080	0.090	0.216	0.197	0.163	0.174	0.194	0.174
1085.869	0.087	0.085	0.210	0.186	0.203	0.164	0.199	0.164
1112.074	0.080	0.084	0.168	0.184	0.196	0.162	0.191	0.162
1408.006	0.069	0.074	0.096	0.163	0.076	0.143	0.124	0.143

Table S4. The μ_m rates of the PS-b-PEG copolymers blended with the nanostructured SeO_2 particles.

**NC Label**	**PSNC1**		**PSNC2**		**PSNC3**		**PSNC4**		**PSNC5**	
	$\mu_m\ (cm^2/g)$									
**Energy (keV)**	**EXP.**	**THEO.**	**EXP.**	**THEO.**	**EXP.**	**THEO.**	**EXP.**	**THEO.**	**EXP.**	**THEO.**
121.782	0.235	0.268	0.333	0.277	0.142	0.251	0.260	0.368	0.364	0.379
344.279	0.198	0.188	0.092	0.121	0.124	0.095	0.107	0.122	0.140	0.161
778.904	0.137	0.133	0.107	0.082	0.077	0.063	0.091	0.079	0.108	0.109
964.079	0.098	0.120	0.102	0.074	0.045	0.057	0.088	0.071	0.071	0.098
1085.869	0.092	0.114	0.077	0.070	0.071	0.053	0.073	0.067	0.072	0.092
1112.074	0.080	0.112	0.087	0.069	0.067	0.053	0.072	0.066	0.075	0.091
1408.006	0.080	0.099	0.062	0.061	0.047	0.047	0.060	0.059	0.045	0.081
**NC Label**	**PSNC6**		**PSNC7**		**PSNC8**		**PSNC9**		**PSNC10**	
	$\mu_m\ (cm^2/g)$									
**Energy (keV)**	**EXP.**	**THEO.**	**EXP.**	**THEO.**	**EXP.**	**THEO.**	**EXP.**	**THEO.**	**EXP.**	**THEO.**
121.782	0.283	0.251	0.228	0.244	0.222	0.376	0.268	0.579	0.229	0.339
344.279	0.194	0.176	0.142	0.107	0.155	0.142	0.180	0.191	0.178	0.144
778.904	0.141	0.125	0.051	0.072	0.072	0.095	0.093	0.125	0.099	0.097
964.079	0.111	0.113	0.055	0.065	0.070	0.085	0.058	0.112	0.083	0.088
1085.869	0.086	0.106	0.052	0.062	0.086	0.080	0.127	0.106	0.099	0.083
1112.074	0.068	0.105	0.041	0.061	0.053	0.079	0.069	0.104	0.085	0.082
1408.006	0.062	0.093	0.042	0.054	0.043	0.070	0.043	0.093	0.082	0.072
**NC Label**	**PSNC11**		**PSNC12**		**PSNC13**		**PSNC14**		**PSNC15**	
	$\mu_m\ (cm^2/g)$									
**Energy (keV)**	**EXP.**	**THEO.**	**EXP.**	**THEO.**	**EXP.**	**THEO.**	**EXP.**	**THEO.**	**EXP.**	**THEO.**

121.782	0.177	0.184	0.297	0.399	0.218	0.442	0.263	0.372	0.301	0.417
344.279	0.156	0.129	0.118	0.175	0.234	0.167	0.177	0.123	0.190	0.178
778.904	0.071	0.091	0.140	0.119	0.152	0.111	0.069	0.080	0.087	0.120
964.079	0.077	0.083	0.107	0.107	0.089	0.099	0.062	0.072	0.061	0.108
1085.869	0.062	0.078	0.112	0.101	0.109	0.094	0.081	0.068	0.119	0.102
1112.074	0.057	0.077	0.106	0.100	0.086	0.093	0.073	0.067	0.105	0.101
1408.006	0.044	0.068	0.051	0.088	0.068	0.082	0.075	0.059	0.054	0.089

Table S5. The μ_m rates of the PS-b-PEG copolymers blended with the nanostructured SeO_2 and BN particles.

**NC Label**	**PBSNC1**		**PBSNC2**		**PBSNC3**		**PBSNC4**	
	$\mu_m\ (cm^2/g)$							
**Energy (keV)**	**EXP.**	**THEO.**	**EXP.**	**THEO.**	**EXP.**	**THEO.**	**EXP.**	**THEO.**
121.782	0.293	0.173	0.269	0.348	0.361	0.331	0.389	0.344
344.279	0.070	0.122	0.106	0.131	0.101	0.109	0.116	0.138
778.904	0.072	0.086	0.099	0.087	0.088	0.071	0.102	0.093
964.079	0.064	0.078	0.083	0.078	0.067	0.064	0.077	0.083
1085.869	0.060	0.073	0.087	0.074	0.062	0.060	0.076	0.078
1112.074	0.047	0.073	0.083	0.073	0.056	0.060	0.064	0.077
1408.006	0.030	0.064	0.060	0.064	0.024	0.053	0.037	0.069
**NC Label**	**PBSNC5**		**PBSNC6**		**PBSNC7**		**PBSNC8**	
	$\mu_m\ (cm^2/g)$							
**Energy (keV)**	**EXP.**	**THEO.**	**EXP.**	**THEO.**	**EXP.**	**THEO.**	**EXP.**	**THEO.**
121.782	0.151	0.145	0.242	0.278	0.515	0.556	0.263	0.267
344.279	0.128	0.103	0.149	0.104	0.122	0.183	0.083	0.107
778.904	0.078	0.073	0.062	0.069	0.116	0.119	0.085	0.072
964.079	0.069	0.066	0.063	0.062	0.064	0.108	0.069	0.065
1085.869	0.068	0.062	0.065	0.059	0.118	0.101	0.059	0.061
1112.074	0.066	0.061	0.063	0.058	0.069	0.099	0.045	0.060
1408.006	0.052	0.054	0.044	0.051	0.028	0.088	0.037	0.053
**NC Label**	**PBSNC9**		**PBSNC10**		**PBSNC11**		**PBSNC12**	
	$\mu_m\ (cm^2/g)$							
**Energy (keV)**	**EXP.**	**THEO.**	**EXP.**	**THEO.**	**EXP.**	**THEO.**	**EXP.**	**THEO.**
121.782	0.142	0.156	0.329	0.353	0.405	0.375	0.329	0.293
344.279	0.094	0.108	0.192	0.132	0.081	0.124	0.171	0.117
778.904	0.079	0.076	0.094	0.088	0.081	0.081	0.115	0.079
964.079	0.061	0.069	0.086	0.079	0.068	0.073	0.079	0.071
1085.869	0.067	0.065	0.084	0.074	0.084	0.068	0.081	0.067
1112.074	0.061	0.064	0.067	0.074	0.081	0.067	0.078	0.066
1408.006	0.052	0.057	0.039	0.065	0.032	0.059	0.050	0.058

S2.2. Half-Value Layer (HVL), Tenth Value Layer (TVL), Mean Free Path (MFP), and Radiation Protection Efficiency (RPE)

Table S6. The HVL and TVL rates of the PS-b-PEG copolymers blended with the nanostructured SeO_2 particles.

**NC Label**	**PSNC1**		**PSNC2**		**PSNC3**		**PSNC4**		**PSNC5**	
**Energy (keV)**	**HVL(cm)**	**TVL(cm)**	**HVL(cm)**	**TVL(cm)**	**HVL(cm)**	**TVL(cm)**	**HVL(cm)**	**TVL(cm)**	**HVL(cm)**	**TVL(cm)**
121.782	2.881	9.569	1.245	4.137	1.940	6.445	1.276	4.240	0.916	3.043
344.279	3.421	11.364	4.505	14.964	2.228	7.400	3.109	10.328	2.387	7.928

778.904	4.930	16.376	3.887	12.911	3.601	11.961	3.634	12.072	3.079	10.228
964.079	6.926	23.008	4.080	13.554	6.201	20.600	3.775	12.542	4.762	15.820
1085.869	7.381	24.518	5.393	17.914	3.891	12.927	4.559	15.146	4.634	15.392
1112.074	8.432	28.010	4.780	15.879	4.109	13.649	4.636	15.400	4.499	14.946
1408.006	8.458	28.097	6.713	22.299	5.880	19.532	5.502	18.276	7.367	24.474
**NC Label**	**PSNC6**		**PSNC7**		**PSNC8**		**PSNC9**		**PSNC10**	
**Energy (keV)**	**HVL(cm)**	**TVL(cm)**	**HVL(cm)**	**TVL(cm)**	**HVL(cm)**	**TVL(cm)**	**HVL(cm)**	**TVL(cm)**	**HVL(cm)**	**TVL(cm)**
121.782	2.457	8.162	1.178	3.913	2.042	6.782	1.684	5.594	2.583	8.579
344.279	3.592	11.934	1.897	6.302	2.936	9.754	2.503	8.314	3.324	11.041
778.904	4.929	16.375	5.280	17.540	6.295	20.911	4.866	16.164	5.974	19.844
964.079	6.243	20.739	4.852	16.117	6.468	21.485	7.825	25.994	7.088	23.547
1085.869	8.125	26.990	5.150	17.109	5.293	17.584	3.567	11.851	5.940	19.731
1112.074	10.209	33.913	6.520	21.660	8.607	28.593	6.515	21.643	6.973	23.162
1408.006	11.266	37.424	6.466	21.478	10.599	35.210	10.607	35.234	7.222	23.990
**NC Label**	**PSNC11**		**PSNC12**		**PSNC13**		**PSNC14**		**PSNC15**	
**Energy (keV)**	**HVL(cm)**	**TVL(cm)**	**HVL(cm)**	**TVL(cm)**	**HVL(cm)**	**TVL(cm)**	**HVL(cm)**	**TVL(cm)**	**HVL(cm)**	**TVL(cm)**
121.782	3.563	11.837	1.853	6.157	1.251	4.156	1.422	4.724	1.484	4.930
344.279	4.032	13.393	4.647	15.436	1.169	3.882	2.113	7.018	2.348	7.800
778.904	8.936	29.683	3.928	13.047	1.791	5.949	5.439	18.068	5.167	17.165
964.079	8.146	27.061	5.158	17.136	3.060	10.167	6.000	19.933	7.280	24.185
1085.869	10.177	33.808	4.901	16.281	2.511	8.341	4.632	15.387	3.738	12.418
1112.074	11.104	36.886	5.185	17.226	3.172	10.535	5.127	17.032	4.254	14.132
1408.006	14.223	47.249	10.825	35.960	3.982	13.229	4.996	16.597	8.274	27.484

Table S7. The HVL and TVL rates of the PS-b-PEG copolymers blended with the nanostructured SeO_2 and BN particles.

**NC Label**	**PBSNC1**		**PBSNC2**		**PBSNC3**		**PBSNC4**	
**Energy (keV)**	**HVL(cm)**	**TVL(cm)**	**HVL(cm)**	**TVL(cm)**	**HVL(cm)**	**TVL(cm)**	**HVL(cm)**	**TVL(cm)**
121.782	1.917	6.367	1.008	3.348	0.746	2.478	0.908	3.015
344.279	7.976	26.495	2.563	8.513	2.660	8.835	3.048	10.125
778.904	7.807	25.936	2.749	9.131	3.056	10.153	3.477	11.549
964.079	8.735	29.018	3.280	10.897	4.043	13.429	4.578	15.208
1085.869	9.304	30.909	3.129	10.394	4.355	14.468	4.676	15.533
1112.074	11.866	39.417	3.282	10.902	4.764	15.826	5.540	18.405
1408.006	18.500	61.455	4.498	14.943	11.081	36.809	9.474	31.472
**NC Label**	**PBSNC5**		**PBSNC6**		**PBSNC7**		**PBSNC8**	
**Energy (keV)**	**HVL(cm)**	**TVL(cm)**	**HVL(cm)**	**TVL(cm)**	**HVL(cm)**	**TVL(cm)**	**HVL(cm)**	**TVL(cm)**
121.782	2.662	8.844	1.410	4.685	0.917	3.045	1.298	4.313
344.279	3.150	10.462	2.285	7.592	3.883	12.899	4.115	13.669
778.904	5.171	17.177	5.476	18.190	4.072	13.527	4.028	13.380
964.079	5.849	19.429	5.451	18.107	7.359	24.445	4.956	16.462
1085.869	5.872	19.505	5.280	17.541	3.987	13.245	5.764	19.147
1112.074	6.070	20.165	5.379	17.870	6.840	22.722	7.586	25.199
1408.006	7.801	25.913	7.804	25.925	16.841	55.945	9.128	30.323
**NC Label**	**PBSNC9**		**PBSNC10**		**PBSNC11**		**PBSNC12**	
**Energy (keV)**	**HVL(cm)**	**TVL(cm)**	**HVL(cm)**	**TVL(cm)**	**HVL(cm)**	**TVL(cm)**	**HVL(cm)**	**TVL(cm)**
121.782	3.721	12.359	0.843	2.799	0.712	2.367	0.850	2.847
344.279	5.616	18.656	1.447	4.805	3.551	11.797	1.655	5.498
778.904	6.649	22.086	2.953	9.809	3.567	11.849	2.462	8.180
964.079	8.689	28.863	3.216	10.685	4.251	14.121	3.583	11.903

1085.869	7.957	26.433	3.295	10.946	3.415	11.345	3.468	11.520
1112.074	8.702	28.906	4.135	13.738	3.541	11.764	3.634	12.071
1408.006	10.112	33.592	7.203	23.929	9.074	30.142	5.601	18.606

Table S8. The MFP and RPE rates of the PS-b-PEG copolymers blended with the nanostructured SeO_2 particles.

NC Label	PSNC1		PSNC2		PSNC3		PSNC4		PSNC5	
Energy (keV)	MFP(cm)	RPE(%)	MFP(cm)	RPE(%)	MFP(cm)	RPE(%)	MFP(cm)	RPE(%)	MFP(cm)	RPE(%)
121.782	4.156	10.219	1.797	19.825	2.799	11.216	1.842	15.356	1.322	21.030
344.279	4.935	8.678	6.499	5.926	3.214	9.843	4.485	6.615	3.443	8.663
778.904	7.112	6.105	5.607	6.835	5.195	6.209	5.243	5.687	4.442	6.783
964.079	9.993	4.384	5.887	6.522	8.947	3.654	5.447	5.481	6.871	4.440
1085.869	10.648	4.120	7.780	4.975	5.614	5.759	6.578	4.560	6.685	4.560
1112.074	12.164	3.616	6.896	5.594	5.928	5.463	6.688	4.487	6.491	4.693
1408.006	12.203	3.605	9.685	4.017	8.483	3.850	7.937	3.794	10.629	2.893
**NC Label**	**PSNC6**		**PSNC7**		**PSNC8**		**PSNC9**		**PSNC10**	
**Energy (keV)**	**MFP(cm)**	**RPE(%)**	**MFP(cm)**	**RPE(%)**	**MFP(cm)**	**RPE(%)**	**MFP(cm)**	**RPE(%)**	**MFP(cm)**	**RPE(%)**
121.782	3.545	12.960	1.699	15.788	2.946	11.654	2.430	10.333	3.726	15.239
344.279	5.183	9.056	2.737	10.119	4.236	8.256	3.611	7.076	4.795	12.056
778.904	7.111	6.685	7.618	3.761	9.082	3.940	7.020	3.705	8.618	6.898
964.079	9.007	5.316	6.999	4.086	9.331	3.836	11.289	2.320	10.226	5.846
1085.869	11.722	4.111	7.430	3.854	7.637	4.667	5.147	5.019	8.569	6.936
1112.074	14.728	3.285	9.407	3.057	12.418	2.897	9.400	2.780	10.059	5.940
1408.006	16.253	2.982	9.328	3.082	15.291	2.359	15.302	1.717	10.419	5.741
**NC Label**	**PSNC11**		**PSNC12**		**PSNC13**		**PSNC14**		**PSNC15**	
**Energy (keV)**	**MFP(cm)**	**RPE(%)**	**MFP(cm)**	**RPE(%)**	**MFP(cm)**	**RPE(%)**	**MFP(cm)**	**RPE(%)**	**MFP(cm)**	**RPE(%)**
121.782	5.141	11.241	2.674	12.760	1.805	9.842	2.052	15.314	2.141	16.223
344.279	5.816	10.003	6.704	5.299	1.686	10.498	3.048	10.585	3.387	10.585
778.904	12.891	4.644	5.666	6.238	2.584	6.982	7.847	4.253	7.455	4.957
964.079	11.752	5.082	7.442	4.786	4.415	4.147	8.657	3.863	10.503	3.544
1085.869	14.683	4.089	7.071	5.031	3.623	5.031	6.683	4.975	5.393	6.787
1112.074	16.019	3.754	7.481	4.762	4.576	4.005	7.397	4.505	6.138	5.988
1408.006	20.520	2.943	15.617	2.310	5.745	3.202	7.208	4.621	11.936	3.125

Table S9. The MFP and RPE rates of the PS-b-PEG copolymers blended with the nanostructured SeO_2 and BN particles.

NC Label	PBSNC1		PBSNC2		PBSNC3		PBSNC4	
Energy (keV)	MFP(cm)	RPE(%)	MFP(cm)	RPE(%)	MFP(cm)	RPE(%)	MFP(cm)	RPE(%)
121.782	2.765	17.293	1.454	14.690	1.076	22.334	1.310	22.334
344.279	11.507	4.460	3.697	6.057	3.837	6.843	4.397	7.251
778.904	11.264	4.554	3.965	5.659	4.409	5.982	5.016	6.386
964.079	12.602	4.080	4.733	4.764	5.832	4.557	6.605	4.888
1085.869	13.424	3.836	4.514	4.989	6.283	4.237	6.746	4.788
1112.074	17.119	3.020	4.735	4.762	6.873	3.880	7.993	4.056
1408.006	26.689	1.948	6.490	3.497	15.986	1.687	13.668	2.393
**NC Label**	**PBSNC5**		**PBSNC6**		**PBSNC7**		**PBSNC8**	
**Energy (keV)**	**MFP(cm)**	**RPE(%)**	**MFP(cm)**	**RPE(%)**	**MFP(cm)**	**RPE(%)**	**MFP(cm)**	**RPE(%)**
121.782	3.841	10.894	2.035	16.341	1.322	19.388	1.873	19.744
344.279	4.544	9.290	3.297	10.425	5.602	4.960	5.936	6.705
778.904	7.460	5.766	7.900	4.491	5.875	4.736	5.811	6.845

964.079	8.438	5.115	7.864	4.511	10.616	2.649	7.150	5.600
1085.869	8.471	5.095	7.618	4.653	5.752	4.834	8.315	4.834
1112.074	8.757	4.933	7.761	4.570	9.868	2.847	10.944	3.695
1408.006	11.254	3.860	11.259	3.173	24.297	1.166	13.169	3.080
**NC Label**	**PBSNC9**		**PBSNC10**		**PBSNC11**		**PBSNC12**	
**Energy (keV)**	**MFP(cm)**	**RPE(%)**	**MFP(cm)**	**RPE(%)**	**MFP(cm)**	**RPE(%)**	**MFP(cm)**	**RPE(%)**
121.782	5.368	9.890	1.216	16.827	1.028	21.669	1.237	21.986
344.279	8.102	6.667	2.087	10.178	5.123	4.781	2.388	12.066
778.904	9.592	5.661	4.260	5.122	5.146	4.761	3.553	8.279
964.079	12.535	4.362	4.640	4.713	6.133	4.010	5.169	5.766
1085.869	11.480	4.753	4.754	4.603	4.927	4.967	5.003	5.952
1112.074	12.554	4.355	5.966	3.685	5.109	4.794	5.242	5.688
1408.006	14.589	3.759	10.392	2.132	13.091	1.899	8.081	3.728

S3. Characterization Examinations of Polymer-Nanostructured Particles Based Nanocomposites via SEM System

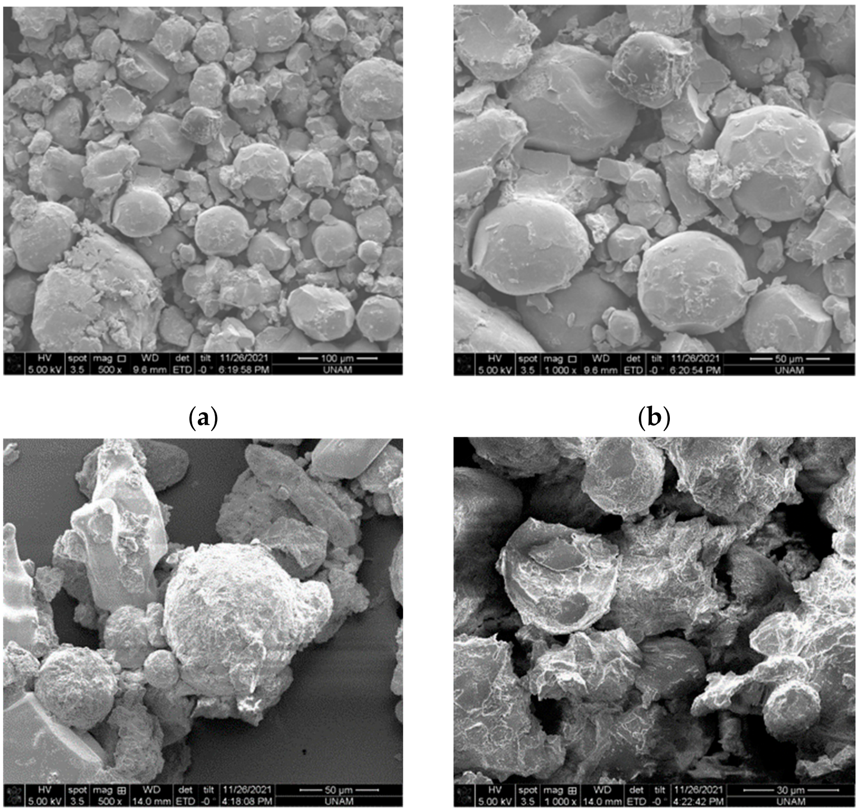

(**a**) (**b**) (**c**) (**d**)

Figure S1. SEM images of PSNC1 nanocomposite (**a-b**) and PSNC4 (**c-d**) nanocomposites.

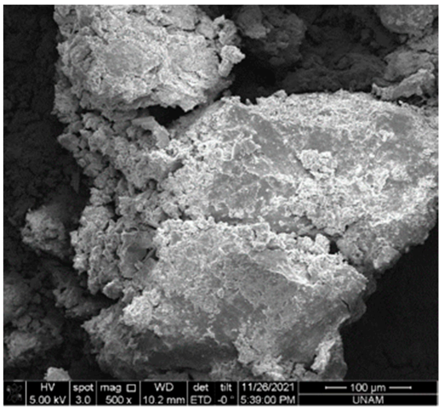
HV spot mag WD det tilt 11/26/2021
5.00 kV 3.0 500 x 10.2 mm ETD -0 ° 5:39:00 PM
100 µm
UNAM

(**a**)

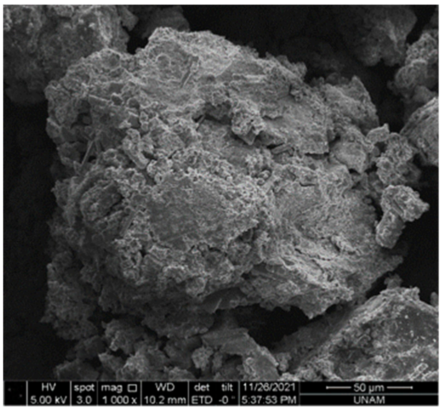
HV spot mag WD det tilt 11/26/2021
5.00 kV 3.0 1 000 x 10.2 mm ETD -0 ° 5:37:53 PM
50 µm
UNAM

(**b**)

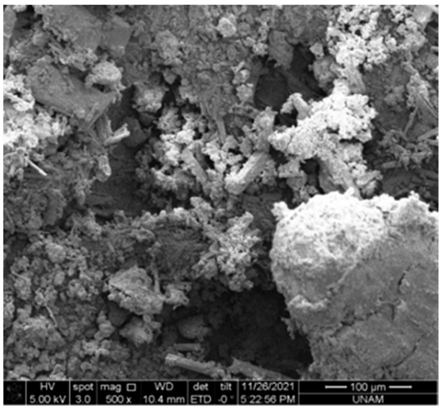
HV spot mag WD det tilt 11/26/2021
5.00 kV 3.0 500 x 10.4 mm ETD -0 ° 5:22:56 PM
100 µm
UNAM

(**c**)

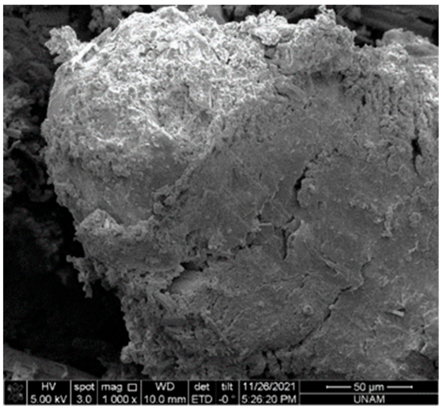
HV spot mag WD det tilt 11/26/2021
5.00 kV 3.0 1 000 x 10.0 mm ETD -0 ° 5:26:20 PM
50 µm
UNAM

(**d**)

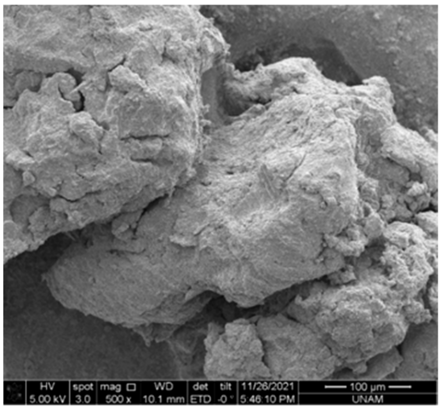
HV spot mag WD det tilt 11/26/2021
5.00 kV 3.0 500 x 10.1 mm ETD -0 ° 5:46:10 PM
100 µm
UNAM

(**e**)

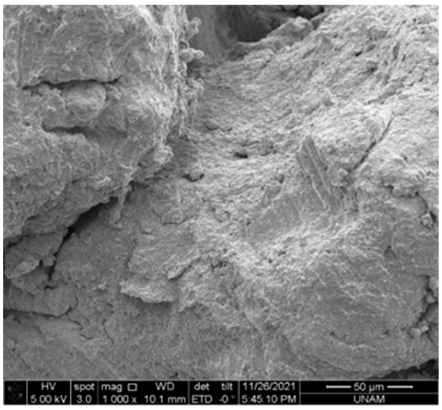
HV spot mag WD det tilt 11/26/2021
5.00 kV 3.0 1 000 x 10.1 mm ETD -0 ° 5:45:10 PM
50 µm
UNAM

(**f**)

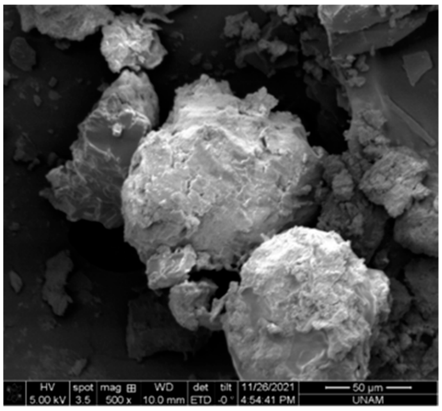
HV spot mag WD det tilt 11/26/2021
5.00 kV 3.5 500 x 10.0 mm ETD -0 ° 4:54:41 PM
50 µm
UNAM

(**g**)

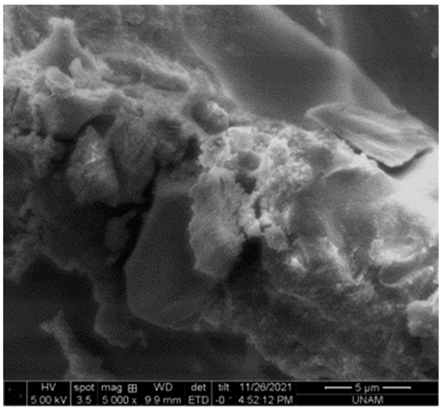
HV spot mag WD det tilt 11/26/2021
5.00 kV 3.5 5 000 x 9.9 mm ETD -0 ° 4:52:12 PM
5 µm
UNAM

(**h**)

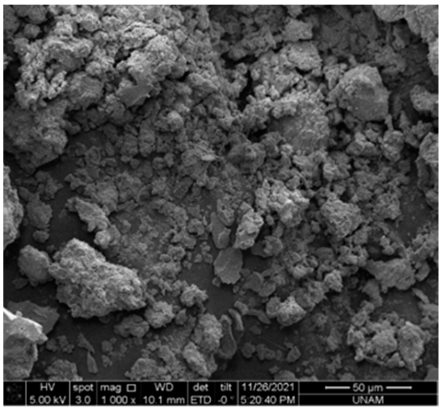

(**i**)

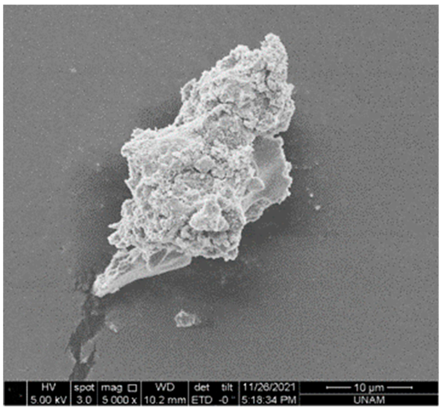

(**j**)

Figure S2. SEM images of PSNC9 (**a-b**), PSNC14 (**c-d**), PBSNC2 (**e-f**), PBSNC6 (**g-h**), and PBSNC10 (**i-j**) nanocomposites.
